# Testing for functional significance of traits: Effect of the light environment in tropical tree saplings

**DOI:** 10.1002/ece3.7499

**Published:** 2021-04-02

**Authors:** Guilherme Silva Modolo, Victor Alexandre Hardt Ferreira dos Santos, Marciel José Ferreira

**Affiliations:** ^1^ Department of Forest Sciences Federal University of Amazonas Manaus Brazil; ^2^ Departamento de Engenharia Florestal Centro de Estudos Superiores de Itacoatiara Universidade do Estado do Amazonas Itacoatiara Brazil

**Keywords:** Central Amazon, functional traits, growth, intraspecific variation, irradiance, photosynthesis, plant strategies

## Abstract

1. Functional traits have been examined to explain the growth rates of forest communities in different sites. However, weak or nonexistent relations are often found, especially due to the following methodological aspects: 1) lack of an environmental context (e.g., light, water, or nutrient supply), 2) use of nonfunctional traits, 3) an approach that does not contemplate phenotypic integration, and 4) neglect of intraspecific variation.
2. Here we measured relative growth rates, crown, and leaf traits in saplings of six tropical tree species growing in two light environments (Gap and Understory) to test whether contrasting light environments modulates trait–trait and trait–growth relationships. Moreover, we tested whether models that integrate traits of different dimensions of the plant (crown and leaf) improve the strength of trait–growth relations.
3. Light availability changed both trait–trait and trait–growth relationships. Overall, in Understory, crown traits (crown length and total leaf area) have a stronger effect on growth rates, while physiological traits related to nutrient acquisition (nitrogen concentration), photochemical efficiency (chlorophyll pigments and chlorophyll *a* fluorescence), and biochemical efficiency (potassium use efficiency) are strong in Gap. Models including multiple traits explained growth rates better in Gap (up to 62%) and Understory (up to 47%), but just in Gap the best model comprises traits that are representative of different dimensions of the plant.
4. *Synthesis*. We advanced the knowledge behind the light effects on tree sapling by posit that trait–trait and trait–growth relationships vary across light environments. Therefore, light availability is a key environmental factor to be considered when choosing the set of traits to be measured in functional approach studies using tropical tree saplings. In compliance with the phenotype integration hypothesis, functional traits are better predictors of growth rates when grouped in a set of traits of different dimensions of the plant that represent different functional mechanisms.

## Graphical abstract

Light availability changes both trait–trait and trait–growth relations.Crown traits have a stronger effect on growth rates in understory.Physiological traits have a stronger effect on growth rates in gap.

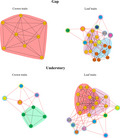

## 1 INTRODUCTION

Functional traits are by definition any morphological, physiological, or phenological measurements performed at an individual level that indirectly affect fitness due to effects on some aspect of plant performance (i.e., growth, survival, or reproduction) (Violle et al., 2007). Ecologists have long used traits to explain demography performance affected by abiotic and biotic environmental conditions around the world, especially growth rate (Chave et al., 2009; Paine et al., 2015; Wright et al., 2004). However, studies that found a moderate or strong trait–growth rate relationship are an exception (Swenson et al., 2020). In this sense, recent studies have concentrated efforts in understanding why weak relationships are frequent and defining standardized protocols for studies of trait‐based community ecology (Caruso et al., 2020; Swenson et al., 2020; Worthy & Swenson, 2019; Yang et al., 2018). Some methodological aspects of sampling may affect the ability of traits to predict growth rates, such as 1) lack of environmental context, 2) use of nonfunctional traits, 3) approaches that do not contemplate phenotypic integration, and 4) neglect of intraspecific variation (Yang et al., 2018; Yang et al., 2020; Worthy & Swenson, 2019).

To understand how traits determine plant growth, it is necessary to understand how traits correlate to form plant phenotype and how environmental context modulates these relationships (He et al., 2020; McGill et al., 2006). Globally, trait–trait relationships were ordered in a single axis of variation, where species with high specific leaf area, high photosynthetic rate, high nitrogen content, and low wood density (among other traits) have acquisitive strategies and a high growth, while the opposite trend reflects conservative strategies and low growth (Chave et al., 2009; Reich, 2014; Wright et al., 2004). However, these trends of covariation may change among environments (Nicotra et al., 1997; Poorter et al., 2014; Wright et al., 2005), from global to local scale (Messier et al., 2017) and from interspecific to intraspecific analysis (Laughlin et al., 2017). For example, a maximum photosynthetic rate (*A*_max_) increases positively according to specific leaf area (*SLA*) among species (Wright et al., 2004), but across a light gradient the intraspecific correlation is negative, as acclimation of leaves to high light increases *A*_max_ but reduces *SLA* (Kenzo et al., 2015; Santos & Ferreira, 2020b). Furthermore, trait–growth relationships may also change according to light environment (Poorter, 1999). For example, in high‐light environments, physiological traits related to photosynthetic nutrient use efficiency are highly related to growth (Guimarães et al., 2018), while in low‐light environments, morphological traits related to light interception are highly related to growth (Liu et al., 2016). Studies that examine the effects of environmental contexts on trait–trait and trait–growth relationships are necessary, especially in tropical forests (Worthy & Swenson, 2019).

Beyond the environmental context, a good selection of traits is fundamental. The traits commonly measured and the type of analyses conducted represent little the complexity of the growth process. Most studies developed to date have focused on measuring soft traits due to an easy applicability and a lower required investment (Gibert et al., 2016; Worthy & Swenson, 2019; Yang et al., 2018). However, hard traits are more informative of physiological processes that drive plant performance because they highlight, for example, the different steps of photosynthesis (Santos & Ferreira, 2020b; Volaire et al., 2020; Worthy & Swenson, 2019; Yang et al., 2018). Additionally, a set of traits can better explain demographic rates than a single trait does (Li et al., 2017) because plant growth depends on a complex integration of multiple traits. Therefore, models that allow integration of many traits measured in different dimensions of the plant, such as crown, leaf, roots, and stem (the phenotypic integration hypothesis), that represent mechanisms related to demographically complex processes, such as growth, may have a greater explanation power (Yang et al., 2020).

The great variation in outcomes found in the literature on functional trait–demographic rate relations is one of the most important topics in plant biology (Salguero‐Gómez et al., 2018). Considering light, the most limiting factor to plant growth in tropical forests (Goldstein et al., 2016; Graham et al., 2003; Nicotra et al., 1999; Wagner et al., 2017), the role the availability of this resource plays in trait–growth relationship remains unclear. Thus, to fill this gap, an analysis that controls light variation and species composition can better demonstrate how traits determine the performance of plants (Wright et al., 2010). Therefore, based on a two‐year experiment of secondary forest enrichment planting with six tropical tree species introduced in artificial gaps, we selected individuals growing in contrasting light environments (Gap and Understory) and then measured thirty traits (from crown and leaf) at an individual level. We analyzed the relationships among traits and between traits and growth rate and tested the following three questions. First, how does light environment modulate trait–trait relationships in tropical tree saplings? We hypothesize that the plant phenotype is manifested by different trait‐to‐trait relationships at the different light environment. Second, does the light environment modulate the relationships between traits and growth rate? We hypothesize that light environment changes the plant phenotype in a way (first hypothesis) that affects traits most related to growth. Third, do trait–growth relationships improve by using an integrated set of traits, measured in different dimensions of the plant (crown and leaf)? We hypothesize that a set of traits of different dimensions of plants—the whole phenotype hypothesis—and physiological traits (e.g., photosynthetic process) represents growth better in both environments.

## 2 MATERIAL AND METHODS

### 2.1 Study site

This study was conducted in a secondary forest enrichment planting. In 1997, the forest was cut and burned to implement a *Theobroma gradiflorum* (Willd. ex Spreng.) K. Schum. (cupuaçu tree) crop. However, the area was abandoned, giving way to a naturally regenerating forest. Therefore, silvicultural treatments were applied during the last quarter of 2016 to begin the enrichment planting experiment in a 19‐year secondary forest. The site is at the Fazenda Experimental da Universidade Federal do Amazonas (02º38'S, 60º03.5'W), 38 km north of Manaus, Amazonas, Brazil. The annual rainfall in Manaus is 2,350 mm, the monthly average air temperature ranges from 26.4 to 28.5ºC, and the average air humidity ranges from 75% in the dry season to 85% in the wet season (data from 1988–2018; INMET, 2019). Rainfall seasonality is generally moderate, with a dry season (rainfall <100 mm) between August and September (Sombroek, 2001).

### 2.2 Experimental design

The light manipulation from the secondary forest to the subsequently enrichment planting consisted of *understory slashing*: removal of all herbaceous plants and small trees (DBH < 5 cm) at the beginning of the experiment and twice a year, and *canopy refinement*: bringing trees down (DBH ≥ 5 cm) by the canopy and subcanopy with a chainsaw in progressive levels of basal area reduction (0, 20, 40, 60, 80, and 100%). Each level of canopy refinement was applied to a plot of 2,318 m^2^ (61 × 38 m), and understory slashing was applied in a subplot half the size of the main canopy refinement plot (Figure S1). Treatment combinations were replicated five times (blocks), each with 12,768 m^2^ (114 × 112 m). Six tropical tree species were selected by the criteria of economic and social importance, life history strategy, and availability of seeds following a minimum number of five matrices. The species are of two ecological groups: long‐lived pioneer (*Cedrela fissilis*, *Tabebuia rosea*, and *Swietenia macrophylla*), and partial shade‐tolerant (*Bertholletia excelsa*, *Carapa guianensis*, and *Hymenaea courbaril*) (Chazdon, 2014; Finegan, 1992; Poorter et al., 2006; Swaine & Whitmore, 1988). A preview study within the same experiment showed a wide range of growth responses of these species to a light gradient created by thinning (Santos & Ferreira, 2020a). The seedlings were planted in these plots in March 2017. In each subplot, five seedlings of each species were planted, 3 × 3 m apart, with an edge of 10 m between plots and 11 m between subplots (Figure S1). During planting, the soil surrounding each seedling was fertilized with P_2_O_5_ (46 g), N (11.6 g), KCl (12 g), and micronutrients (10 g of FTE‐BR12: 1.8% B; 0.8% Cu; 3.0% Fe; 2.0% Mn; 0.1% Mo). The application methods and doses that ensured the minimal nutrient requirements of tropical trees were obtained from a literature review (Alvarado, 2015; Campoe et al., 2014; Furtini Neto et al., 2000; Resende et al., 2005). Further details are provided in Santos et al. (2020) and Santos and Ferreira (2020a).

For this study, we selected only two subplot combinations with the most contrasting light environments: 1) clear‐cut (100% of canopy refinement) and understory slashed subplots—hereinafter named Gap (photosynthetic photon flux density—PPFD = 27.9 mol/m^2^ day^−1^), and 2) plots without basal area reduction (0% of canopy refinement) combined with understory slashing—hereinafter named Understory (PPFD = 1.3 mol/m^2^ day^−1^) (Santos et al., 2020) (Figure S1). We selected only subplots with understory slashed with the objective to reduce the eventual effects of herbaceous competition on sapling growth.

### 2.3 Growth rate measurements

We monitored saplings planted in this experiment for two years in bi‐monthly campaigns from March 2017 to March 2019. In each monitoring campaign, we measured the root collar diameter (*D*) (5 cm above the soil) and total height (*H*) of each sapling. The aboveground biomass was approximated by *D* ^2^ *H*, as Kohyama and Hotta (1990) proposed. We calculated annual relative growth in biomass for each individual according to Hunt (1978).

$$RG_{x}=\frac{\text{ln}\;X_{2}‐\;\text{ln}X_{1}}{T_{2}‐T_{1}}$$

where *RG_x_* = relative growth in biomass, ln*X*_2_ − ln*X*_1_ = increase in logarithmic biomass between two measurements, and *T*_2_–*T*_1_ = interval between measurements.

### 2.4 Trait measurements

We measured a set of crown and leaf traits at the end of the second year after planting (Table 2). We measure all live individuals in each plot of each treatment selected and used individuals values for all analyses. In total, we measured the traits in 113 individuals in Gap, ranging from 16 to 23 per species, and 89 individuals in Understory subplots, ranging from seven to 22 per species. The differences in sampling between light conditions and across species are due the greater mortality of individuals in Understory, especially of shade‐intolerant species (long‐lived pioneers). All leaf traits were measured on healthy, fully expanded leaves visually without herbivory in the middle third of the crown. We grouped traits in two main groups (crown and leaf) and leaf traits in six sets of traits: leaf display, nutrient acquisition, light absorption efficiency, light use efficiency, gas exchange, and nutrient use efficiency.

### 2.5 Crown

We measured crown diameter in two orthogonal directions and crown length (*CL*) from the first leaf insertion (or branch) to the top of the crown. Then, we calculated mean crown diameter (*MCD*, average of two orthogonal crown diameters), crown projection area (*CPA*, using the formula for ellipse area), crown ratio (*CR*, *MCD*/*D*), crown length ratio (*CLR*, *CL/H*), and relative crown length (*RCL*, *CL/MCD*) (Li et al., 2017).

We estimated total leaf area (*TLA*) as follows: First, we counted the numbers of leaves in each plant and measured leaf length and width in the field for 30% of leaves. Then, we used leaf‐size and species‐specific models that relate leaf area to leaf length and width to estimate the area of leaves measured in the field (length and width). The parameters of the models were adjusted using leaves previously measured in laboratory by a leaf area meter (CI‐202, CID, Inc. Beds). The coefficient of determination (*R^2^*) of models ranged from 0.86 to 0.99 (Table S1). Finally, we estimated the total leaf area of each plant using the proportion of leaf area in 30% of leaves. From *TLA*, we also calculated leaf area index (*LAI*) as the ratio between *TLA* and *CPA* (Poorter, 1999).

### 2.6 Leaf display

We measured the leaf area of a range of three to six leaves for each individual using a leaf area meter (CI‐202, CID, Inc. Beds) and then calculated the average to obtain the individual leaf area of each plant (*LA*). Ten leaf disks (2.83 cm^2^) were cut from three leaves per plant, saturated in distillated water, and oven‐dried at 65ºC. Specific leaf area (*SLA*) was calculated as the ratio between fresh leaf area (cm^2^) and oven‐dried mass (g). Leaf dry matter content (*LDMC*) was calculated as the ratio between oven‐dried mass (mg) and water‐saturated mass (g) (Pérez‐Harguindeguy et al., 2013).

### 2.7 Nutrient acquisition

We estimated mass‐based nitrogen, phosphorus, and potassium concentration in three to six leaves per plant. Leaf nitrogen (*N*_mass_) was determined by the Kjeldahl method using digestion, distillation, and titration (Bremner, 1996). Leaf phosphorus (*P*_mass_) was determined by spectrophotometry (λ = 750 nm) following the molybdate method (Murphy & Riley, 1962). Leaf potassium (*K*_mass_) was obtained by atomic absorption spectrometry (1100B, PerkinElmer).

### 2.8 Light absorption efficiency

We estimated mass‐based chloroplast pigment concentrations in three leaves of each plant. Chlorophyll *a* (*Chl a*), chlorophyll *b* (*Chl b*), and carotenoids (*Car_c+x_*) concentrations were extracted with acetone (10 ml of 80% acetone with 0.05 g of MgCO_3_ per 0.1 g of fresh leaf) followed by filtration (Lichtenthaler & Wellburn, 1983). The concentrations were calculated by absorbance reading at three wavelengths (663, 645, and 480 nm; Biochrom Libra s50 UV/Vis) following Hendry and Price (1993). Total chlorophyll concentration (*Chl a* + *b*) and chlorophyll *a* and *b* ratio (*Chl a*/*b*) were calculated by sum and ratio of *Chl a* and *Chl b* concentrations, respectively.

### 2.9 Light use efficiency

The fluorescence parameters were measured in twelve leaves per plant between 8 a.m. and 10 a.m. using a portable fluorimeter (PEA, MK2‐9600, Hansatech) adjusted to emit a saturating light pulse of 3,000 µmol/m^2^ s^−1^ at a wavelength of 650 nm for 1 s. Before the measurements, the leaves were acclimated to the dark during 30 min for a complete oxidation of the photosynthetic electron transport chain. Maximum quantum yield of PSII (*F*_V_/*F*_M_), ABS‐based performance index (*PI*_ABS_), and total performance index (*PI*_total_) were calculated by performing JIP test applied to chlorophyll *a* polyphasic transient stages (Strasser et al., 1995; Strasser et al., 1999; Strasser et al., 2010; Tsimilli‐Michael & Strasser, 2008). These parameters have been widely used in studies to show the responses of plants to abiotic and biotic stresses and light use efficiency on the photosynthetic process (Santos & Ferreira, 2020b; Santos et al., 2019). The JIP test calculates light use performance parameters throughout the electron transport chain. According to this method, light energy is absorbed (ABS) by the antenna of the photosystem II (PSII), and a fraction is trapped (TR) by open PSII reaction centers, leading to quinone A reduction (QA). The QA electron is transported to intersystem electron acceptors (ET) and to the final electron acceptors (RE) of the photosystem I (PSI). The following parameters using the JIP test are obtained: reaction center density (RC/ABS), maximum quantum yield of PSII (*F*_V_/*F*_M_), efficiency of intersystem transport (ET_0_/TR_0_), and efficiency of the electron transport in reducing the final electron acceptor of PSI (RE_0_/ET_0_). *PI*_ABS_ is an integrated index of the efficiency at which electron is trapped by the PSII (*F*_V_/*F*_M_) and transferred further than the QA (ET_0_/TR_0_).

$$PI_{\mathrm{ABS}}=\left(\frac{\mathrm{RC}}{\mathrm{ABS}}\right)*\left(\frac{F_{V}/F_{M}}{1‐F_{V}/F_{M}}\right)*\left(\frac{{\text{ET}}_{0}/{\text{TR}}_{0}}{1‐{\text{ET}}_{0}/{\text{TR}}_{0}}\right).$$

*PI*_total_ is an integrated index of the *PI*_ABS_ and the efficiency at which electrons reduce the end acceptors in the PSI.

$$PI_{\text{total}}=PI_{\text{ABS}}*\left(\frac{{\text{ET}}_{0}/{\text{TR}}_{0}}{1‐{\text{ET}}_{0}/{\text{TR}}_{0}}\right).$$

### 2.10 Gas exchange

We measured leaf gas exchange on one leaf per plant using an infrared gas analyzer (IRGA’s, LI‐6400XT, LI‐COR). Prior to recording, tests were performed on at least three leaves per plant to ensure the selection of leaves with the maximum values of *A*_max_ and *g*_s_. The measurements were made between 8 a.m. and 11 a.m. The IRGA’s chamber was adjusted to a flow rate of 400 µmol/s, CO_2_ concentration of 400 µmol/mol, H_2_O vapor concentration of 21 mmol/mol, leaf temperature of 31ºC, and photosynthetic photon flux density (PPFD) of 2,000 µmol/m^2^ s^−1^ for maximum photosynthetic rate (*A*_max_), stomatal conductance (*g*_s_), and transpiration rate (*E*), and 0 µmol/m^2^ s^−1^ for respiration rate in the dark (*R*_d_).

### 2.11 Nutrient use efficiency

We calculated carbon use efficiency (*CUE*) as the ratio between *A*_max_ and *R*_d_, and nitrogen (*PNUE*), phosphorus (*PPUE*), and potassium (*PKUE*) use efficiency as the ratios between maximum photosynthetic rate per unit mass (*A*_max_ × *SLA* × 0.1) and the nutrient concentrations in molar mass units.

### 2.12 Statistical analyses

We assessed trait and growth rate variation in the 5th and 95th percentile of each light environment (Gap and Understory). To evaluate whether trait–trait relationships changed between light environments, we performed plant trait network analyses (PTNs; He et al., 2020) derived from the Pearson correlation matrix (*p* < 0.05) (Tables S2 and S3) for each light environment and trait level (crown and leaf) separately. In PTNs, complex relationships among traits are explained by the calculation of parameters, which enables an improved view of plant adaptation in response to the changes in resource availability. For each PTNs, we calculated edge density (ED), modules, and hub traits. ED is the ratio of actual connections among traits in relation to the total possible connections and reflects the complexity of the network. Networks with a high ED reflect a stronger coordination among multiple traits. Modules are clusters formed by traits that show covariation among themselves, relatively independently from other clusters. Biologically, modules are a grouping of traits that reflect the functional mechanisms of plants. Hub traits are those with a higher number of actual connections in the network in relation to all possible connections. These traits play a central regulatory role, probably affecting the entire phenotype. For details of PTNs construction and parameters calculation, see He et al. (2020).

To evaluate whether trait–growth relationships were modulated by light environment, we used an individual‐level analysis with traits measured on the same individuals that they were used to predict growth. We adjusted mixed effects models with growth rate as the dependent variable, traits as the independent variable, light environment as the independent categorical variable, and we tested species and plots as random effects. We performed this analysis for each trait separately. We observed interaction effects on relationships with a significance level (*p* < 0.05). When we observe significant interaction effects on relations, we compare the slopes between light environments and showed uncertainty around the estimates. The best models (random effects definition) were selected based on the Akaike's information criterion (AIC). In all models, we observed the coefficients of determination both marginal (for fixed effects) and conditional (for fixed and random effects). Logarithmic transformations were performed to meet linearity and normality of assumptions whenever necessary.

To investigate whether trait–growth rate relationships improved when performed models that integrate traits of different dimensions of the plant, we adjusted mixed effect models with growth as the dependent variable and a set of traits as the independent variable for each light environment. In all models, we tested species and plot as random effects. We performed this analysis with original and composite traits. Composite traits were derived from PCA axes scores. First, we performed a principal component analysis (PCA) for all traits (general PCA). However, when traits had a high correlation or represented a similar biological mechanism, we selected only one of them. In the final calculation, we used 19 traits for the general PCA (Figure S2). Then, we used the scores of general PCA axes that jointly explained most of the variation (~60%) as independent variables in the models. Secondly, we performed PCA for traits of each group (crown, leaf display, nutrients acquisition, light absorption efficiency, light use efficiency, gas exchange, and nutrient use efficiency) separately (categorized PCAs). Next, we used the scores of the first and second axis of each categorized PCA as independent variables in the models. We performed categorized PCAs because they summarize plant mechanisms in a few axes of variation and can improve the explanatory power of models due aggregate the explanation power of many traits, the same idea is applied to general PCA. Finally, we selected the best model for all traits based on the AIC. We also observed the best model with a single trait to compare how much the multiple models improve the power of explanation of growth rates. All traits were standardized, subtracting each individual trait value from the trait mean and then dividing by its standard deviation. Coefficient of determination marginal and significance level (*P* value) were observed to determine the strength of the relations.

The PTNs were fitted using the *igraph* package (He et al., 2020), and mixed effects models were fitted using the *nlme* package (Zuur et al., 2009). All analyses were performed using the statistical software R, version 3.5.1 (R Core Team, 2018).

## 3 RESULTS

### 3.1 Effects of light environment on variations and relations of functional traits

**FIGURE 1:**
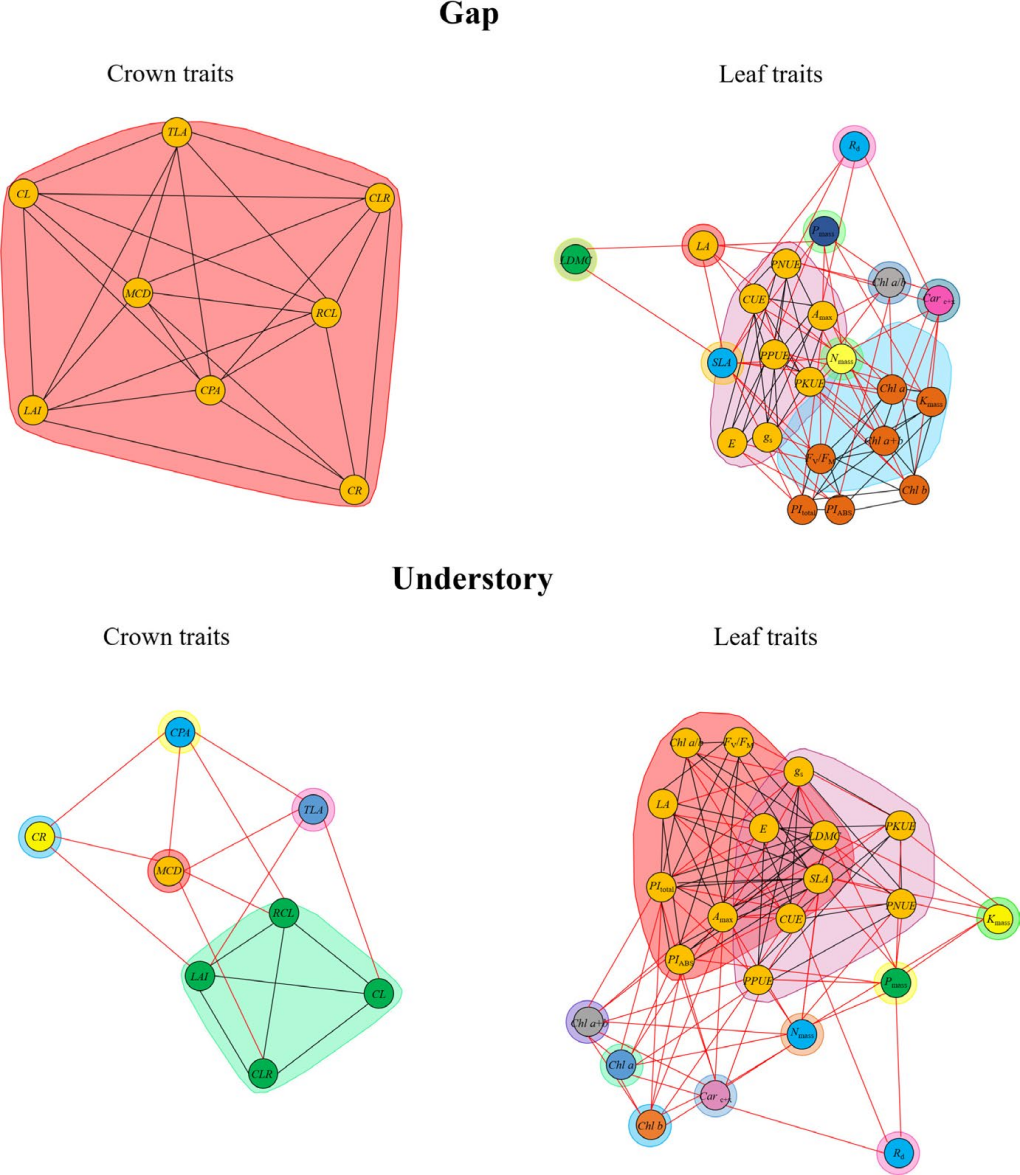
Plant trait network analyses (PTNs) of crown traits and leaf traits in two light environments. Modules that reflect biological mechanisms of plants are indicated by the colors of circles and shaded regions. Lines represent a significant relationship (connection) between traits. Black lines represent the connection between traits of the same module. Red lines represent the connection of traits of different modules. PTNs with a higher number of connections (lines) have a higher edge density. For abbreviations, see Table 1 in the section Results

The range of variation (5th–95th) of growth and traits changes between light environments (Table 1). The PTNs analyses showed that trait–trait relationships were affected by light environment according to overall connectivity, distinction of modules, and traits with a higher connectivity (Figure 1). Overall, ED was higher for crown traits (ED at Gap = 0.89; Understory = 0.61) than for leaf traits (Gap = 0.43; Understory = 0.48). All crown traits were grouped in a same module in Gap, while in Understory traits related to crown depth (*CL*, *CLR*, *RCL,* and *LAI*) formed a distinct module. Regarding leaf traits, both light environments formed two main modules, but in Gap these modules were more distinct. Leaf traits in Gap were grouped in two distinct modules as follows: photochemical efficiency traits (light‐blue shaded area) with predominance of traits related to light absorption efficiency and light use efficiency (except *K*_mass_), and biochemical efficiency traits (purple shaded area) with predominance of traits related to gas exchange and nutrients use efficiency (Figure 1). In Understory, we identified an overlap of modules related to photochemical and biochemical efficiency and the presence of more leaf display traits (Figure 1). Crown traits had a high connectivity among them in both light environments. The ratio of connections of each trait in relation to all possible connections ranged from 75% to 100% in Gap and from 50% to 75% in Understory. Among leaf traits, those with a connectivity greater than 50% were *PKUE* (68%), *A*_max_ (64%), *N*_mass_ (59%), *Chl a* (55%), *F*_V_/*F*_M_ (55%) and *PNUE* (55%) in Gap; *A*_max_ (86%), *PI*_total_ (68%), *PPUE* (68%), *SLA* (64%), *LDMC* (59%), *PI*_ABS_ (59%), *N*_mass_ (55%), *CUE* (55%), and *PNUE* (55%) in Understory.

**TABLE 1:** Name, abbreviation (AB), units, and 5th and 95th percentiles of relative growth rate and thirty measured traits in Gap and Understory

Name	AB	Units	Gap	Understory
Growth rate				
Relative growth rate	RGR	percent year^−1^	1.85–3.46	0.33–1.45
Crown trait				
Mean crown diameter	*MCD*	m	0.71–3.25	0.32–0.99
Crown projection area	*CPA*	m^2^	0.40–8.31	0.08–0.76
Crown length	*CL*	m	0.27–3.53	0.03–1.09
Crown ratio	*CR*		1.67–5.13	2.78–6.97
Crown length ratio	*CLR*		0.11–0.83	0.05–0.89
Relative crown length	*RCL*		0.17–2.15	0.04–1.78
Total leaf area	*TLA*	m^2^	1.02–15.66	0.03–0.45
Leaf area index	*LAI*		0.58–7.20	0.18–0.80
Leaf trait				
Leaf area	*LA*	cm^2^	21.22–156.75	16.89–118.45
Specific leaf area	*SLA*	cm^2^ g^−1^	80.91–137.12	152.32–427.54
Leaf dry matter	*LDMC*	mg/g	256.59–464 0.23	178.06–400.25
Leaf nitrogen	*N*_mass_	g/kg	11.25–21.77	10.23–19.38
Leaf phosphorus	*P*_mass_	g/kg	0.51–1.02	0.60–1.31
Leaf potassium	*K*_mass_	g/kg	4.38–14.84	3.09–13.18
Chlorophyll *a*	*Chl a*	µmol/g	0.56–2.13	0.92–3.08
Chlorophyll *b*	*Chl b*	µmol/g	0.18–0.66	0.36–1.13
Carotenoids	*Car*_c+x_	µmol/g	0.29–0.81	0.37–1.09
Total chlorophyll	*Chl a* + *b*	µmol/g	0.72–2.82	1.26–4.21
Chlorophyll *a* and *b* ratio	*Chl a*/*b*		2.36–3.82	2.32–3.10
Maximum Quantum yield of PSII	*F*_V_/*F*_M_		0.738–0.827	0.814–0.846
ABS‐based performance index	*PI*_ABS_		0.940–4.927	1.217–3.601
Total performance index	*PI*_total_		0.737–2.793	0.324–1.130
Maximum photosynthetic	*A*_max_	µmol CO_2_ m^−2^ s^−1^	7.86–19.49	4.19–11.78
Dark respiration	*R*_d_	µmol CO_2_ m^−2^ s^−1^	0.88–2.64	0.18–1.23
Stomatal conductance	*g*_s_	mol H_2_O m^−2^ s^−1^	0.12–0.53	0.07–0.29
Transpiration rate	*E*	mmol H_2_O m^−2^ s^−1^	2.57–7.50	1.50–4.64
Carbon use efficiency	*CUE*		5.02–16.41	4.95–51.37
Nitrogen use efficiency	*PNUE*	nmol CO_2_ mol^−1^ *N*/m^2^ s^−1^	82.36–191.58	92.44–247.40
Phosphorus use efficiency	*PPUE*	nmol CO_2_ mol^−1^ P m^−2^ s^−1^	3,957.48–10,029.23	2,881.22–10,067.65
Potassium use efficiency	*PKUE*	nmol CO_2_ mol^−1^ K/m^2^ s^−1^	290.57–1,515.12	390.79–1,650.62

### 3.2 Effects of light environment on trait–growth relationships

**FIGURE 2:**
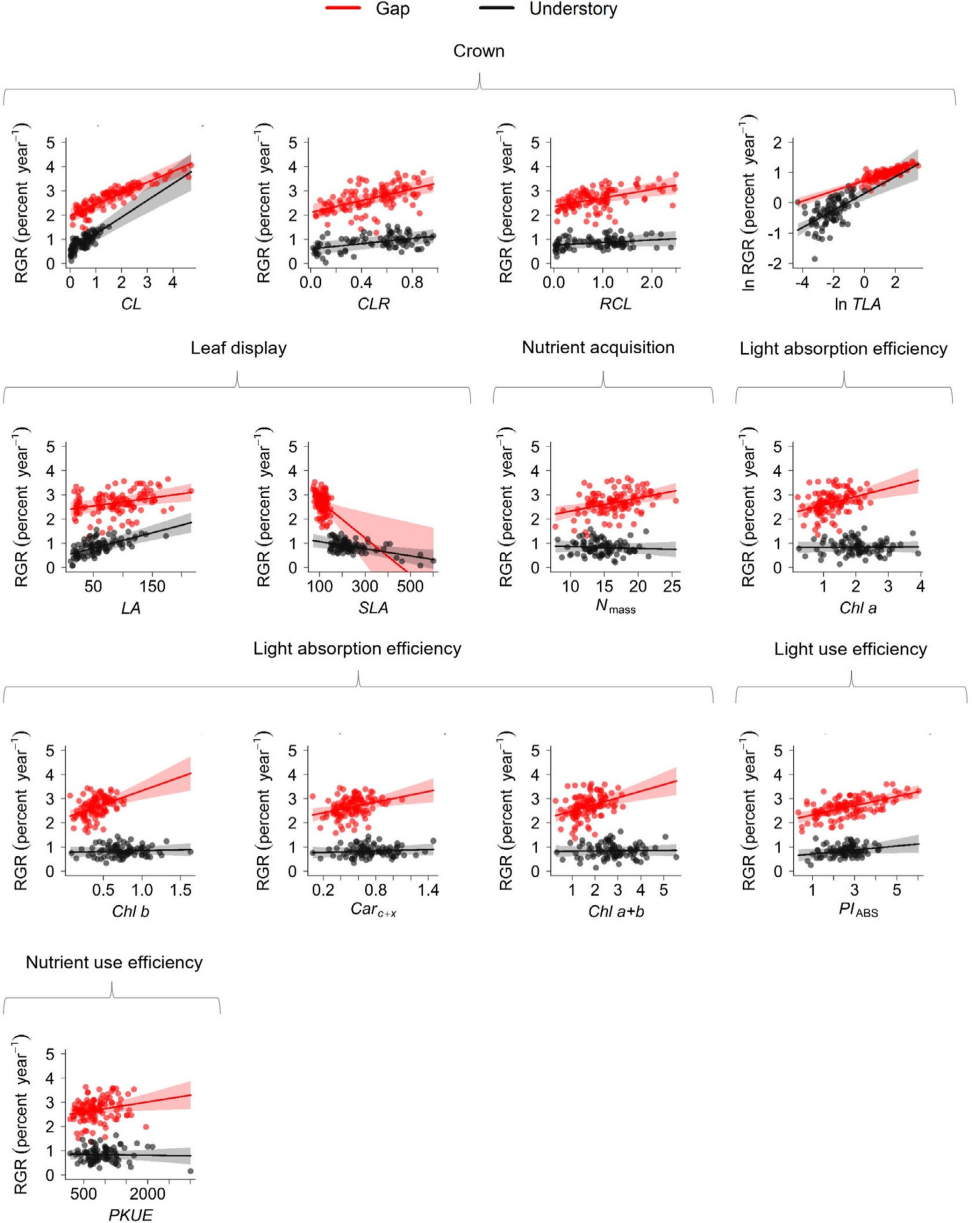
Effect of light environment on trait–growth relationships for traits with significant interaction effects. Points displaying values of growth and trait of individuals. Shading displaying 95% confidence intervals. For abbreviations, see Table 1 in the section Results

Light environments modulated trait–growth rate relationships for some traits, as the significant interaction effects show (Figure 2). Overall, traits related to light interception (*CL* and *TLA*) had higher positive effects on growth rates in Understory than in Gap, with slopes of the relations being 52% to 66% higher. However, for *CLR* and *RCL*, the slope of the relation in Gap was 122% and 233% greater than in Understory, respectively. The positive effects of *LA* on growth rates were high for plants growing in Understory, while the negative effect of *SLA* was greater in Gap (five‐fold). Traits related to nutrient acquisition (*N*_mass_), light absorption efficiency (*Chl a*, *Chl b*, *Car_c_*_+_ *_x_*_,_ and *Chl a* + *b*), light use efficiency (*PI*_ABS_), and photosynthetic nutrient use efficiency (*PKUE*) had higher and positive effects on growth rates in Gap than in Understory, with slopes of relationships being seven to 103 times higher. Table S2 shows the values of slopes and uncertainties.

### 3.3 General and categorized principal component analyses

Most trait variations were explained by the first four axes of the general PCA in both light environments (Figure S2). In the Gap environment, showed by traits with the highest loadings (*L*), the first four axes of general PCA explained 59.5% of trait variation and associated with biochemical efficiency (PC1 = 23.2%; *L* = *A*_max_, *PKUE*, *PPUE* and *PNUE*), leaf structure, nutrient acquisition (PC2 = 15.2%; *L* = *P*_mass_, *N*_mass_, *K*_mass_ and *SLA*), nutrient use efficiency (PC3 = 11.9%; *L = PNUE*), and carbon balance (PC4 = 9.2%; *L* = *R*_d_ and *CUE*). In Understory, the first four axes explained 64.3% of trait variation and associated with biochemical and photochemical efficiency (PC1 = 21.5%; *L* = *A*_max_, *PNUE, PPUE* and *PI*_total_), leaf structure (PC2 = 21.3%; *L* = *SLA* and *LDMC*), nutrient acquisition and use efficiency (PC3 = 11.6%; *L* = *PKUE, K*_mass_ and *P*_mass_), and respiration (9.9%; *L* = *R*_d_). The first axis of the categorized PCAs explained 45.5 (crown) to 74.3% (light absorption efficiency), and the second axis explained 20.3 (light use efficiency) to 37.2% (crown) of trait variation in Gap (Figure S3). In Understory, the first axis of the categorized PCAs explained 41.2 (crown) to 76.6% (light absorption efficiency), and the second axis explained 18.3 (light use efficiency) to 32.5% (crown) of trait variation (Figure S4).

### 3.4 Relationships among growth rates and an integrated set of traits

The combinations of traits and the power of explanation of models changed according to light environments and the set of traits (single, multiple, or multiple with PCA scores, Table 2). First, the best models with a single trait explained 50% of growth variation in Gap (*CL*) and 36% of growth variation in Understory (*MCD*). Secondly, the best multiple traits model including all traits in their original form explained 62% of growth variation at Gap (*MCD* + *CL *+ CR + *PI*_total_) and 47% at Understory (*MCD* + *CL* + CR). Thirdly, the models including the first four axis of general PCA explained 41% of growth variation in Gap (PC1 + PC2 + PC3 + PC4) and 5% in Understory (PC1). Finally, the models including the first and second axes of categorized PCAs explained 52% of growth variation in Gap (PC1_crown_ + PC2_crown_ + PC1_light use efficiency_ + PC2_light use efficiency_) and 28% in Understory (PC2_crown_ + PC2_nutrients acquisition_). Therefore, all models explained better growth rates in Gap than in Understory and multiple trait models improved growth prediction, however not using a PCA score approach.

**TABLE 2:** Results of mixed effect models examining the relations between growth rates and an integrated set of traits and between growth rates in both light environments

Set of traits	Model	*R^2^*_m_	AIC
Gap			
Single trait	(0.985***) *CL*	0.50	209.05
Multiple traits	(0.665***) *MCD* + (0.390***) *CL* + (−0.612***) *CR* + (0.161**) *PI*_total_	0.62	158.71
Four first axis of general PCA	(−0.440***) PC1 + (0.292**) PC2 + (−0.552***) PC3 + (−0.194*) PC4	0.41	252.26
First and second axis of categorized PCAs	(−0.716***) PC1_crown_ + (−0.226*) PC2_crown_ + (−0.213**) PC1_light use efficiency_ + (0.177**) PC2_light use efficiency_	0.52	215.69
Understory			
Single trait	(0.656***) *MCD*	0.36	188.56
Multiple traits	(0.727***) *MCD* + (0.304**) *CL* + (−0.428***) *CR*	0.47	155.84
Four first axis of general PCA	(−0.226*) PC1	0.05	230.23
First and second axis of categorized PCAs	(0.597***) PC2_crown_ + (−0.174*) PC2_nutrients acquisition_	0.28	186.24

Notes

Random effects of the best models are controlled for intercepts of species. Coefficient of determination marginal (*R^2^*_m_) of relationships. *P* values of traits in the best models are * *p* < 0.05, ** *p* < 0.01, *** *p* < 0.001, not significant (n.s.). *p* ≥ 0.05. For abbreviations, see Table 1 in the section Results.

## 4 DISCUSSION

The relationships between functional traits and demographic rates consist in the main framework of trait‐based plant ecology. They are a major challenge in studies on tropical tree ecology (Chave et al., 2009; Wright et al., 2004, 2010). However, ecologists have reported many traits as poor predictors of tree growth, leading to questioning whether these traits are indeed functional or not (Poorter et al., 2018; Swenson et al., 2020; Worthy & Swenson, 2019; Yang et al., 2018). Three core reasons have been stated for the lack of success of traits in determining demographic outcomes in trait‐based functional approach ecology: frequently ignored contextual information (e.g., environmental gradients), functional processes (e.g., photosynthesis) that determine plant performance are not completely explained using only soft traits or a single trait, and focus on species relative to individuals (Worthy & Swenson, 2019; Yang et al., 2018). Here we combined multiple morphophysiological traits and provided evidence on the importance of including the environment context in studies of trait‐based community ecology due its role in modulating the trait–growth relationships of tropical tree saplings. Moreover, we reinforce that growth is the result of the expression of many traits, but may be decoupled of an integrated plant phenotypic expression in light‐limited environments. Even though our results are for six species, these species show a wide variation in the whole plant economic spectrum (Reich, 2014), as values of traits show (Table 1).

### 4.1 Relations among traits change according to light environment

Light environment affected relationships among traits and consequently integrated phenotype (Figure 1). Growing in Gap, all plant crown traits were coordinated and formed a single module, while in Understory only crown traits related to crown depth formed a dense module. Crown depth in Understory can reflect a conservative mechanism because we observed that plants with deep crowns had older leaves inserted at the lowest stem height and some since planting. Plants growing in poor environments show a long leaf lifespan and tend to be more successful in surviving because they compensate for the high payback times needed to return carbon investments on leaf construction (Kitajima & Poorter, 2010; Poorter & Bongers, 2006; Poorter et al., 2006). Evidently, photochemical and biochemical efficiency traits were grouped into two distinct modules in Gap. However, these modules are similar and have many connected traits, proving that photochemical and biochemical efficiency are strongly coordinated (Chou et al., 2020; Santos et al., 2019). Traits related to biochemical and photosynthetic nutrient use efficiency were “hub traits” in both light environments. However, *LDMC* and *SLA* had a high connectivity (>50%) only in the network of Understory. Therefore, photosynthetic efficiency is key in both Understory and Gap; however, in a light‐limited understory, a conservative strategy related to physically structured and durable leaves for protection against herbivores and long‐term carbon investment also play a role (Kitajima, 1994; Kitajima & Poorter, 2010).

### 4.2 Relationships between growth rates and functional traits are modulated by light environment

Overall, in Understory, light interception traits (*LA*, *CL,* and *TLA*) play a more important role in growth than in the Gap (Figure 2). The disproportional effects of light foraging traits are expected in more limited light environments and have been reported as a strategy to optimize the interception of diffuse light or sun flecks reaching the forest floor (Delagrange et al., 2006; Iida et al., 2014; Niklas, 1989). On the other hand, traits representing mechanisms of nutrients acquisition (*N*_mass_), photochemical efficiency (*Chl a*, *Chl b*, *Car_c_*_+_ *_x_* *Chl a* + *b*, *PI*_ABS_), and biochemical efficiency (*PKUE*) more strongly affected the growth rates in Gap than in Understory. Plants growing in Gap have a greater amount of light energy for use in the physiological process (Chazdon & Fetcher, 1984). In this sense, it is more important for saplings to have a high potential for processing this energy in Gap than in Understory (Figure 2). Our results support the hypothesis that the relationships between functional traits and growth rates of tropical tree saplings depend on the environmental context (Worthy & Swenson, 2019) and specifically light environment.

### 4.3 Growth rates are better explained by a multiple set of traits

Our results sustain the hypothesis that an integrated set of traits measured in different dimensions of the plant better explains plant growth. Moreover, the set of traits related to growth and the strength of relations changed between contrasting light environments (Table 2). Growth models that include multiple traits of different dimensions of the plant in the original form of traits or in composite traits better explain growth rates because they allow performing a phenotypic integration, that is, the integration of multiple traits that form the phenotype (Yang et al., 2020). The selection of traits in different plant organs (e.g., crown, leaf, stem, branches, and roots), which are related to different mechanisms that affect the growth process, is interesting for phenotypic integration, making it possible to have a broad view of the main factors that determine plant growth (Rosas et al., 2019; Swenson et al., 2020; Wright et al., 2005; Yang et al., 2020). The production ecology model (PEM) theory states that plant productivity depends on the availability of natural resources and on the efficiency of plant in acquiring and using those resources (Binkley et al., 2004; Monteith, 1977). Thus, measuring traits related to the different stages of PEM is important to understand the factors that determine the differential performance of plants between light environments. Light interception traits at a crown level (*MCD*, *CL,* and *CR*) were included in the best models in both Gap and Understory, but one light use trait (*PI*_total_) was also present in the best model for Gap. Therefore, an integrated approach with multiple trait models improves the growth prediction in the sense that more mechanisms are represented in high and low‐light environments.

Although composite traits (such as those summarized on a PCA axis) could be potential predictors of growth rates (Liu et al., 2016; Yu et al., 2020), in our study they did not capture the variation of the main processes related to growth, mainly in a light‐limited environment (Table 2). The main leaf traits (hubs) observed in plant network traits did not compose the best models in both environments. In Understory, the best multiple trait model comprised no leaf trait. This result may be interpreted as a decoupling between the integrated phenotype expression of leaf traits and growth performance. In this sense, the mechanisms behind leaf traits could be expressed as a strategy to maximize another component of plant fitness (e.g., sapling survival), mainly in a light‐limited environment that demands conservative strategies (Kunstler et al., 2016; Wright et al., 2010). Moreover, the strength of relationships was higher for Gap than for Understory (Table 2). These results corroborate with the hypothesis Poorter et al. (2018) proposed. The authors stated that low‐light availability harms the expression of growth and traits, resulting in a convergence of these values and hence weak or nonexistent relationships.

### 4.4 Implications for functional approach studies

Gap dynamics is one of the main factors that leads to biodiversity in tropical forests. However, to investigate demographic rates in the gap dynamics is expensive and time‐consuming (Chazdon, 2014; Connell, 1978). Thereby, the use of functional traits to uncover mechanisms that control the dynamics of forest communities following appropriate methodologies is an option for quick results in ecology studies (Salguero‐Gómez et al., 2018; Swenson et al., 2020; Worthy & Swenson, 2019; Yang et al., 2018). We add knowledge to this functional approach by showing that a complex process such as growth is better represented by multiple trait models, with traits measured in different dimensions of the plant. Thus, future studies measuring traits in different plant organs (i.e., crown, leaf, stem, branches, and roots) that are related to different processes of plant growth can provide a broader view of factors that determine plant growth. Following this approach, our results also highlight the importance in considering the environmental context (e.g., light supply) during the selection of traits to be measured. Traits measured at crown level are more associated with growth rates of saplings in low‐light environments but are also good predictors of growth rates in high‐light environments. Nevertheless, *PI*_total_, an index of the entire photochemical efficiency accessed by chlorophyll *a* fluorescence, is a good predictor of growth rates in a high‐light environment. Chlorophyll *a* fluorescence parameters are related to photochemical stage of photosynthesis and have been considered a proxy of carbon assimilation in tropical trees along the vertical profile of Central Amazonian forest (Santos et al., 2019). Therefore, chlorophyll *a* fluorescence parameters should be incorporated in functional studies because, in addition to its power to explain sapling growth, it also represents a fast and noninvasive method. Finally, due to the capacity of plants to achieve similar performances with alternative designs of traits (Dias et al., 2020; Marks & Lechowicz, 2006; Worthy et al., 2020), future studies should consider the intraspecific trait–growth relation of each species and/or ecologic group.

## 5 CONCLUSIONS

This study provides evidences on the importance of considering the environmental context (light supply) when deciding which traits to measure in future functional ecological researches. Functional traits seem to be better predictors of growth performance in high‐light environments, especially when grouped in multiple traits. Multiple trait measurements in different dimensions of the plant that represent different processes of plant growth can contribute significantly to key ecological questions related to the main mechanisms behind tropical forest dynamics.

## CONFLICT OF INTEREST

None declared.

## AUTHORS CONTRIBUTIONS

**Guilherme Modolo:** Conceptualization (equal); Formal analysis (lead); Writing‐original draft (lead); Writing‐review & editing (equal). **Victor Alexandre dos Santos:** Conceptualization (equal); Formal analysis (equal); Methodology (equal); Supervision (equal); Writing‐review & editing (equal). **Marciel Ferreira:** Conceptualization (equal); Funding acquisition (lead); Investigation (equal); Methodology (equal); Project administration (lead); Resources (lead); Supervision (equal); Writing‐review & editing‐(equal).

## Supporting information

Supplementary Material

## Data Availability

Data are publicly accessible at the Mendeley Data. http://dx.doi.org/10.17632/6thg4w3vdm.1

## References

[ece37499-bib-0001] Alvarado, A. (2015). Plant nutrition in tropical forestry. In L. Pancel & M. Köhl (Eds.), Tropical forestry handbook (pp. 1114–1182). Springer. 10.1007/978-3-642-54601-3_105

[ece37499-bib-0002] Binkley, D. , Stape, J. L. , & Ryan, M. G. (2004). Thinking about efficiency of resources use in forests. Forest and Ecology Management, 193, 5–16. 10.1016/j.foreco.2004.01.019

[ece37499-bib-0003] Bremner, J. M. (1996). Nitrogen‐total. In D. l. Sparks , A. l. Page , P. A. Helmke , R. H. Loeppert , P. N. Soltanpour , M. A. Tabatabai , C. T. Johnston , & M. E. Sumner (Eds.), Methods of soil analysis (pp. 1085–1121). Soil Science Society of America, American Society of Agronomy. 10.2136/sssabookser5.3.c37

[ece37499-bib-0004] Campoe, O. C. , Iannelli, C. , Stape, J. L. , Cook, R. L. , Mendes, J. C. T. , & Vivian, R. (2014). Atlantic forest tree species responses to silvicultural practices in a degraded pasture restoration plantation: From leaf physiology to survival and initial growth. Forest Ecology and Management, 313, 233–242. 10.1016/j.foreco.2013.11.016

[ece37499-bib-0005] Caruso, C. M. , Manson, C. M. , & Medeiros, J. S. (2020). The evolution of functional traits in plants: In the giant still sleeping? International Journal of Plant Science, 181, 1–8. 10.1086/707141

[ece37499-bib-0006] Chave, J. , Coomes, D. , Jansen, S. , Lewis, S. L. , Swenson, N. G. , & Zanne, A. E. (2009). Towards a worldwide wood economics spectrum. Ecology Letters, 12, 351–366. 10.1111/j.1461-0248.2009.01285.x 19243406

[ece37499-bib-0007] Chazdon, R. L. (2014). Second growth: The promise of tropical forest regeneration in an age of deforestation. University of Chicago Press. 10.7208/chicago/9780226118109.001.0001

[ece37499-bib-0008] Chazdon, R. L. & Fetcher, N. (1984). Photosynthetic light environments in a lowland tropical rain forest in Costa Rica. Journal of Ecology, 72, 553–564. 10.2307/2260066

[ece37499-bib-0009] Chou, S. , Chen, B. , Chen, J. , Wang, M. , Wang, S. , Croft, H. , & Shi, Q. (2020). Estimation of leaf photosynthetic capacity from the photochemical reflectance index and leaf pigments. Ecological Indicators, 110, 105867. 10.1016/j.ecolind.2019.105867

[ece37499-bib-0010] Connell, J. H. (1978). Diversity in tropical rain forests and coral reefs. Science, 199, 1302–1310. 10.1126/science.199.4335.1302 17840770

[ece37499-bib-0011] Delagrange, S. , Montpied, P. , Dreyer, E. , Messier, C. , & Sinoquet, H. (2006). Does shade improve light interception efficiency? A comparison among seedlings from shade‐tolerant and ‐intolerant temperate deciduous tree species. New Phytologist, 172, 293–304. 10.1111/j.1469-8137.2006.01814.x 16995917

[ece37499-bib-0012] Dias, A. T. C. , Rosado, B. H. P. , De Bello, F. , Pistón, N. , & De Mattos, E. A. (2020). Alternative plant designs: Consequences for community assembly and ecosystem functioning. Annals of Botany, 125, 391–398. 10.1093/aob/mcz180 31678986 PMC7442394

[ece37499-bib-0013] Finegan, B. (1992). The management potential of neotropical secondary lowland rain forest. Forest Ecology and Management, 47, 295–321. 10.1016/0378-1127(92)90281-D

[ece37499-bib-0014] Furtini Neto, A. E. , Siqueira, J. O. , Curi, N. , & Moreira, F. M. (2000). Fertilização em reflorestamento com espécies nativas. In J. L. M. Gonçalves , & V. Benedetti (Eds.), Nutrição e fertilização florestal (pp. 352–383). IPEF.

[ece37499-bib-0015] Gibert, A. , Gray, E. F. , Westoby, M. , Wright, I. J. , & Falster, D. S. (2016). On the link between functional traits and growth rate: Meta‐analysis shows effects change with plant size, as predicted. Journal of Ecology, 104, 1488–1503. 10.1111/1365-2745.12594

[ece37499-bib-0016] Goldstein, G. , Santiago, L. S. , Campanello, P. I. , Avalos, G. , Zhang, Y.‐J. , & Villagra, M. (2016). Facing shortage or excessive light: How tropical and subtropical trees adjust their photosynthetic behavior and life history traits to a dynamic forest environment. In G. Goldstein & E. L. S. Santiago (Ed.), Tropical tree physiology (pp. 319–336). Springer International Publishing.

[ece37499-bib-0017] Graham, E. A. , Mulkey, S. S. , Kitajima, K. , Phillips, N. G. , & Wright, J. (2003). Cloud cover limits net CO_2_ uptake and growth of a rainforest tree during tropical rainy seasons. Proceedings of the National Academy of Sciences, 100, 572–576. 10.1073/pnas.0133045100 PMC141037 12518044

[ece37499-bib-0018] Guimarães, Z. T. M. , dos Santos, V. A. H. F. , Nogueira, W. L. P. , Martins, N. O. A. , & Ferreira, M. J. (2018). Leaf traits explaining the growth of tree species planted in a Central Amazonian disturbed area. Forest Ecology and Management, 430, 618–628. 10.1016/j.foreco.2018.08.048

[ece37499-bib-0019] He, N. , Li, Y. , Liu, C. , Xu, L. , Li, M. , Zhang, J. , He, J. , Tang, Z. , Han, X. , Ye, Q. , Xiao, C. , Yu, Q. , Liu, S. , Sun, W. , Niu, S. , Li, S. , Sack, L. , & Yu, G. (2020). Plant trait networks: Improved resolution of the dimensionality of adaptation. Trends in Ecology & Evolution, 35, 908–918. 10.1016/j.tree.2020.06.003 32595068

[ece37499-bib-0020] Hendry, G. A. F. , & Price, A. H. (1993). Stress indicators: Chlorophylls and carotenoids. In G. A. F. Hendry , & J. P. Grime (Eds.), Methods in comparative plant ecology (pp. 148–152). Chapman & Hall.

[ece37499-bib-0021] Hunt, R. (1978). Plant growth analysis. Studies in Biology No. 96 (p. 37). Edward Arnold.

[ece37499-bib-0022] Iida, Y. , Poorter, L. , Sterck, F. , Kassim, R. , Potts, M. D. , Kubo, T. , & Kohyama, T. S. (2014). Linking size‐dependent growth and mortality with architectural traits across 145 co‐occurring tropical tree species. Ecology, 95, 353–363. 10.1890/11-2173.1 24669729

[ece37499-bib-0023] Instituto Nacional de Meteorologia . (2019). BDMEP—Banco de Dados Meteorológicos para Ensino e Pesquisa. https://portal.inmet.gov.br/dadoshistoricos accessed 20 June 2019

[ece37499-bib-0024] Kenzo, T. , Inoue, Y. , Yoshimura, M. , Yamashita, M. , Tanaka‐Oda, A. , & Ichie, T. (2015). Height‐related changes in leaf photosynthetic traits in diverse Bornean tropical rain forest trees. Oecologia, 177, 191–202. 10.1007/s00442-014-3126-0 25362582

[ece37499-bib-0025] Kitajima, K. (1994). Relative importance of photosynthetic traits and allocation patterns as correlates of seedling shade tolerance of 13 tropical trees. Oecologia, 98, 419–428. 10.1007/BF00324232 28313920

[ece37499-bib-0026] Kitajima, K. & Poorter, L. (2010). Tissue‐level leaf toughness, but not lamina thickness predicts sapling leaf lifespan and shade tolerance of tropical tree species. New Phytologist, 186, 708–721. 10.1111/j.1469-8137.2010.03212.x 20298481

[ece37499-bib-0027] Kohyama, T. & Hotta, M. (1990). Significance of allometry in tropical saplings. Functional Ecology, 4, 515–521. 10.2307/2389319

[ece37499-bib-0028] Kunstler, G. , Falster, D. , Coomes, D. A. , Hui, F. , Kooyman, R. M. , Laughlin, D. C. , Poorter, L. , Vanderwel, M. , Vieilledent, G. , Wright, S. J. , Aiba, M. , Baraloto, C. , Caspersen, J. , Cornelissen, J. H. C. , Gourlet‐Fleury, S. , Hanewinkel, M. , Herault, B. , Kattge, J. , Kurokawa, H. , … Westoby, M. (2016). Plant functional traits have globally consistent effects on competition. Nature, 529, 204–207. 10.1038/nature16476 26700807

[ece37499-bib-0029] Laughlin, D. C. , Lusk, C. H. , Bellingham, P. J. , Burslem, D. F. R. P. , Simpson, A. H. , & Kramer‐Walter, K. R. (2017). Intraspecific trait variation can weaken interspecific trait correlations when assessing the whole‐plant economic spectrum. Ecology and Evolution, 7, 8936–8949. 10.1002/ece3.3447 29152189 PMC5677476

[ece37499-bib-0030] Li, Y. , Kröber, W. , Bruelheide, H. , Härdtle, W. , & von Oheimb, G. (2017). Crown and leaf traits as predictors of subtropical tree sapling growth rates. Journal of Plant Ecology, 10, 136–145. 10.1093/jpe/rtw041

[ece37499-bib-0031] Lichtenthaler, H. & Wellburn, A. (1983). Determinations of total carotenoids and chlorophylls a and b of leaf extracts in different solvents. Biochemical Society Transactions, 11, 591–592. 10.1042/bst0110591

[ece37499-bib-0032] Liu, X. , Swenson, N. G. , Lin, D. , Mi, X. , Umanã, M. N. , Schimid, B. , & Ma, K. (2016). Linking individual‐level functional traits to tree growth in a subtropical forest. Ecology, 97, 2396–2405. 10.1002/ecy.1445 27859093

[ece37499-bib-0033] Marks, C. O. & Lechowicz, M. J. (2006). Alternative designs and the evolution of functional diversity. The American Naturalist, 167, 55–66. 10.1086/498276 16475099

[ece37499-bib-0034] McGill, B. J. , Enquist, B. J. , Weiher, E. , & Westoby, M. (2006). Rebuilding community ecology from functional traits. Trends in Ecology and Evolution, 21, 178–185. 10.1016/j.tree.2006.02.002 16701083

[ece37499-bib-0035] Messier, J. , Lechowicz, M. J. , McGill, B. J. , Violle, C. , & Enquist, B. J. (2017). Interspecific integration of trait dimensions at local scales: The plant phenotype as an integrated network. Journal of Ecology, 105, 1775–1790. 10.1111/1365-2745.12755

[ece37499-bib-0036] Monteith, J. L. (1977). Climate and the efficiency of crop production in Britain. Philosophical Transactions of the Royal Society B: Biological Sciences, 281, 277–294. 10.1098/rstb.1977.0140

[ece37499-bib-0037] Murphy, J. A. M. E. S. & Riley, J. P. (1962). A modified single solution method for the determination of phosphate in natural waters. Analytica Chimica Acta, 27, 31–36. 10.1016/S0003-2670(00)88444-5

[ece37499-bib-0038] Nicotra, A. B. , Chazdon, R. L. , & Iriarte, V. B. (1999). Spatial heterogeneity of light and woody seedling regeneration in tropical wet forests. Ecology, 80, 1908–1926. 10.1890/0012-9658

[ece37499-bib-0039] Nicotra, A. B. , Chazdon, R. L. , & Schlichting, C. (1997). Patterns of genotypic variation and phenotypic plasticity of light response in two tropical *Piper* (Piperaceae) species. American Journal of Botany, 84, 1542–1552. 10.2307/2446616 21708557

[ece37499-bib-0040] Niklas, K. J. (1989). The effect of leaf‐lobing on the interception of direct solar radiation. Oecologia, 80, 59–64. 10.1007/BF00789932 23494346

[ece37499-bib-0041] Paine, C. E. T. , Amissah, L. , Auge, H. , Baraloto, C. , Baruffol, M. , Bourland, N. , Bruelheide, H. , Daïnou, K. , de Gouvenain, R. C. , Doucet, J‐L. , Doust, S. , Fine, P. V. A. , Fortunel, C. , Haase, J. , Holl, K. D. , Jactel, H. , Li, X. , Kitajima, K. , Koricheva, J. , … Hector, A. (2015). Globally, functional traits are weak predictors of juvenile tree growth, and we do not know why. Journal of Ecology, 103, 978–989. 10.1111/1365-2745.12401

[ece37499-bib-0042] Pérez‐Harguindeguy, N. , Díaz, S. , Garnier, E. , Lavorel, S. , Poorter, H. , Jaureguiberry, P. , Bret‐Harte, M. S , Cornwell, W. K , Craine, J. M , Gurvich, D. E , Urcelay, C. , Veneklaas, E. J , Reich, P. B , Poorter, L. , Wright, I. J , Ray, P. , Enrico, L. , Pausas, J. G , de Vos, A. C , … Cornelissen, J. H. C. (2013). New handbook for standardised measurement of plant functional traits worldwide. Australian Journal of Botany, 61, 167–234. 10.1071/BT12225

[ece37499-bib-0043] Poorter, H. , Lambers, H. , & Evans, J. R. (2014). Trait correlation networks: A whole‐plant perspective on the recently criticized leaf economic spectrum. New Phytologist, 201, 378–382. 10.1111/nph.12547 24117716

[ece37499-bib-0044] Poorter, L. (1999). Growth responses of 15 rain‐forest tree species to a light gradient: The relative importance of morphological and physiological traits. Functional Ecology, 13, 396–410. 10.1046/j.1365-2435.1999.00332.x

[ece37499-bib-0045] Poorter, L. & Bongers, F. (2006). Leaf traits are good predictors of plant performance across 53 rain forest species. Ecology, 87, 1733–1743. 10.1890/0012-9658 16922323

[ece37499-bib-0046] Poorter, L. , Bongers, L. , & Bongers, F. (2006). Architecture of 54 moist‐forest tree species: Traits, trade‐offs, and functional groups. Ecology, 87, 1289–1301. 10.1890/0012-9658 16761607

[ece37499-bib-0047] Poorter, L. , Castilho, C. V. , Schietti, J. , Oliveira, R. S. , & Costa, F. R. C. (2018). Can traits predict individual growth performance? A test in a hyperdiverse tropical forest. New Phytologist, 19, 109–121. 10.1111/nph.15206 PMC6001574 29774944

[ece37499-bib-0048] R Core Team . (2018). R: A language and environment for statistical computing. R Foundation for Statistical Computing.

[ece37499-bib-0049] Reich, P. B. (2014). The world‐wide ‘fast–slow’ plant economics spectrum: A traits manifesto. Journal of Ecology, 102, 275–301. 10.1111/1365-2745.12211

[ece37499-bib-0050] Resende, A. V. , Furtini Neto, A. E. , & Curi, N. (2005). Mineral nutrition and fertilization of native tree species in Brazil: Research progress and suggestions for management. Journal of Sustainable Forestry, 20, 45–81. 10.1300/J091v20n02_03

[ece37499-bib-0051] Rosas, T. , Mencuccini, M. , Barba, J. , Cochard, H. , Saura‐Mas, S. , & Martínez‐Vilalta, J. (2019). Adjustments and coordination of hydraulic, leaf and stem traits along a water availability gradient. New Phytologist, 223, 632–646. 10.1111/nph.15684 30636323

[ece37499-bib-0052] Salguero‐Gómez, R. , Violle, C. , Gimenez, O. , & Childs, D. (2018). Delivering the promises of trait‐based approaches to the needs of demographic approaches, and vice versa. Functional Ecology, 32, 1424–1435. 10.1111/1365-2435.13148 30034074 PMC6049886

[ece37499-bib-0053] Santos, V. A. H. F. , & Ferreira, M. J. (2020a). Initial establishment of commercial tree species under enrichment planting in a Central Amazon secondary forest: Effects of silvicultural treatments. Forest Ecology and Management, 460, 117822. 10.1016/j.foreco.2019.117822

[ece37499-bib-0054] Santos, V. A. H. F. & Ferreira, M. J. (2020b). Are photosynthetic leaf traits related to the first‐year growth of tropical tree seedlings? A light‐induced plasticity test in a secondary forest enrichment planting. Forest Ecology and Management, 460, 117900. 10.1016/j.foreco.2020.117900

[ece37499-bib-0055] Santos, V. A. H. F. , Modolo, G. S. , & Ferreira, M. J. (2020). How do silvicultural treatments alter the microclimate in a Central Amazon secondary forest? A focus on light changes. Journal of Environmental Management, 254, 109816. 10.1016/j.jenvman.2019.109816 31743861

[ece37499-bib-0056] Santos, V. A. H. F. , Nelson, B. W. , Rodrigues, J. V. F. C. , Garcia, M. N. , Ceron, J. V. B. , & Ferreira, M. J. (2019). Fluorescence parameters among leaf photosynthesis‐related traits are the best proxies for CO_2_ assimilation in Central Amazon trees. Brazilian Journal of Botany, 42, 239–247. 10.1007/s40415-019-00533-2

[ece37499-bib-0057] Sombroek, W. (2001). Spatial and temporal patterns of Amazon rainfall. Ambio, 30, 388–397. 10.1579/0044-7447-30.7.388 11795213

[ece37499-bib-0058] Strasser, R. J. , Srivastava, A. , & Govindjee, (1995). Polyphasic chlorophyll *a* fluorescence transient in plants and cyanobacteria. Photochemistry and Photobiology, 61, 32–42. 10.1111/j.1751-1097.1995.tb09240.x

[ece37499-bib-0059] Strasser, R. J. , Srivastava, A. , & Tsimilli‐Michael, M. (1999). Screening the vitality and photosynthetic activity of plants by fuorescence transient. In R. K. Behl , M. S. Punia , & B. P. S. Lather (Eds.), Crop improvement for food security (pp. 72–115). SSARM.

[ece37499-bib-0060] Strasser, R. J. , Tsimilli‐Michael, M. , Giang, S. , & Goltsev, V. (2010). Simultaneous in vivo recording of prompt and delayed fluorescence and 820‐nm reflection changes during drying and after rehydration of the resurrection plant *Haberlea rhodopensis* . Biochimica et Biophysica Acta (BBA)—Bioenergetics, 1797(6‐7), 1313–1326. 10.1016/j.bbabio.2010.03.008 20226756

[ece37499-bib-0061] Swaine, M. D. & Whitmore, T. C. (1988). On the definition of ecological species groups in tropical rain forests. Vegetatio, 75, 81–86. 10.1007/BF00044629

[ece37499-bib-0062] Swenson, N. G. , Worthy, S. J. , Eubanks, D. , Iida, Y. , Monks, L. , Petprakob, K. , Rubio, V. E. , Staiger, K. , & Zambrano, J. (2020). A reframing of trait‐demographic rate analyses for ecology and evolutionary biology. International Journal of Plant Sciences, 181, 33–43. 10.1086/706189

[ece37499-bib-0063] Tsimilli‐Michael, M. , & Strasser, R. J. (2008). In vivo assessment of plants’ vitality: Applications in detecting and evaluating the impact of mycorrhization on host plants. In A. Varma (Ed.), Mycorrhiza: State of the art. Genetics and molecular biology, eco function, biotechnology, eco‐physiology, structure and systematics (3rd ed., pp. 679–703). Springer.

[ece37499-bib-0064] Violle, C. , Navas, M. , Vile, D. , Kazakou, E. , Fortunel, C. , Hummel, I. , & Garnier, E. (2007). Let the concepto f trait be functional!. Oikos, 116, 882–892. 10.1111/j.0030-1299.2007.15559.x

[ece37499-bib-0065] Volaire, F. , Gleason, S. M. , & Delzon, S. (2020). What do you mean “functional” in ecology? Patterns versus processes. Ecology and Evolution, 10, 11875–11885. 10.1002/ece3.6781 33209257 PMC7663066

[ece37499-bib-0066] Wagner, F. H. , Hérault, B. , Rossi, V. , Hilker, T. , Maeda, E. E. , Sanchez, A. , Lyapustin, A. I. , Galvão, L. S. , Wang, Y. , & Aragão, L. O. C. (2017). Climate drivers of the Amazon forest greening. PLoS One, 12, 1–15. 10.1371/journal.pone.0180932 PMC5510836 28708897

[ece37499-bib-0067] Worthy, S. J. , Laughlin, D. C. , Zambrano, J. , Umaña, M. N. , Zhang, C. , Lin, L. , Cao, M. , & Swenson, N. G. (2020). Alternative designs and tropical tree seedling growth performance landscapes. Ecology, 101, e03007. 10.1002/ecy.3007 32030743

[ece37499-bib-0068] Worthy, S. J. & Swenson, N. G. (2019). Functional perspectives on tropical tree demography and forest dynamics. Ecological Processes, 8, 1–11. 10.1186/s13717-018-0154-4

[ece37499-bib-0069] Wright, I. J. , Reich, P. B. , Cornelissen, J. H. , Falster, D. S. , Garnier, E. , Hikosaka, K. , Lamont, B. B. , Lee, W. , Oleksyn, J. , Osada, N. , Poorter, H. , Villar, R. , Warton, D. I. , & Westoby, M. (2005). Assessing the generality of global leaf trait relationships. New Phytologist, 166, 485–496. 10.1111/j.1469-8137.2005.01349.x 15819912

[ece37499-bib-0070] Wright, I. J. , Reich, P. B. , Westoby, M. , Ackerly, D. D. , Baruch, Z. , Bongers, F. , Cavender‐Bares, J. , Chapin, T. , Cornelissen, J. H. C. , Diemer, M. , Flexas, J. , Garnier, E. , Groom, P. K. , Gulias, J. , Hikosaka, K. , Lamont, B. B. , Lee, T. , Lee, W. , Lusk, C. , … Villar, R. (2004). The worldwide leaf economics spectrum. Nature, 428, 821–827. 10.1038/nature02403 15103368

[ece37499-bib-0071] Wright, S. J. , Kitajima, K. , Kraft, N. J. B. , Reich, P. B. , Wright, I. J. , Bunker, D. E. , Condit, R. , Dalling, J. W. , Davies, S. J. , Díaz, S. , Engelbrecht, B. M. J. , Harms, K. E. , Hubbell, S. P. , Marks, C. O. , Ruiz‐Jaen, M. C. , Salvador, C. M. , & Zanne, A. E. (2010). Functional traits and the growth‐mortality trade‐off in tropical trees. Ecology, 91, 3664–3674. 10.1890/09-2335.1 21302837

[ece37499-bib-0072] Yang, J. , Cao, M. , & Swenson, N. G. (2018). Why functional traits do not predict tree demographic rates. Trends in Ecology & Evolution, 33, 326–336. 10.1016/j.tree.2018.03.003 29605086

[ece37499-bib-0073] Yang, J. , Song, X. , Cao, M. , Deng, X. , Zhang, W. , Yang, X. , & Swenson, N. G. (2020). On the modeling of tropical tree growth: The importance of intraspecific trait variation, non‐linear functions and phenotypic integration. Annals of Botany, mcaa085. 10.1093/aob/mcaa085 PMC7988515 32361752

[ece37499-bib-0074] Yu, R. , Huang, J. , Xu, Y. , Ding, Y. , & Zang, R. (2020). Plant functional niches in forests across four climatic zones: Exploring the periodic table of niches based on plant functional traits. Frontiers in Plant Science, 11, 841. 10.3389/fpls.2020.00841 32625227 PMC7311788

[ece37499-bib-0075] Zuur, A. F. , Ieno, E. N. , Walker, N. , Saveliev, A. A. , & Smith, G. M. (2009). Mixed effects models and extensions in ecology with R. Statistics for Biology and Health. Springer. 10.1086/648138

