# Supplementary material for: Testing for functional significance of traits: Effect of the light environment in tropical tree saplings
Source: Ecol Evol. 2021 Apr 2;11(11):6480–92. doi: 10.1002/ece3.7499 (PMC8207416; doi:10.1002/ece3.7499)
Supplement: Supplementary file 1 — Supplementary Material [file ECE3-11-6480-s001.docx]

# Supplementary material


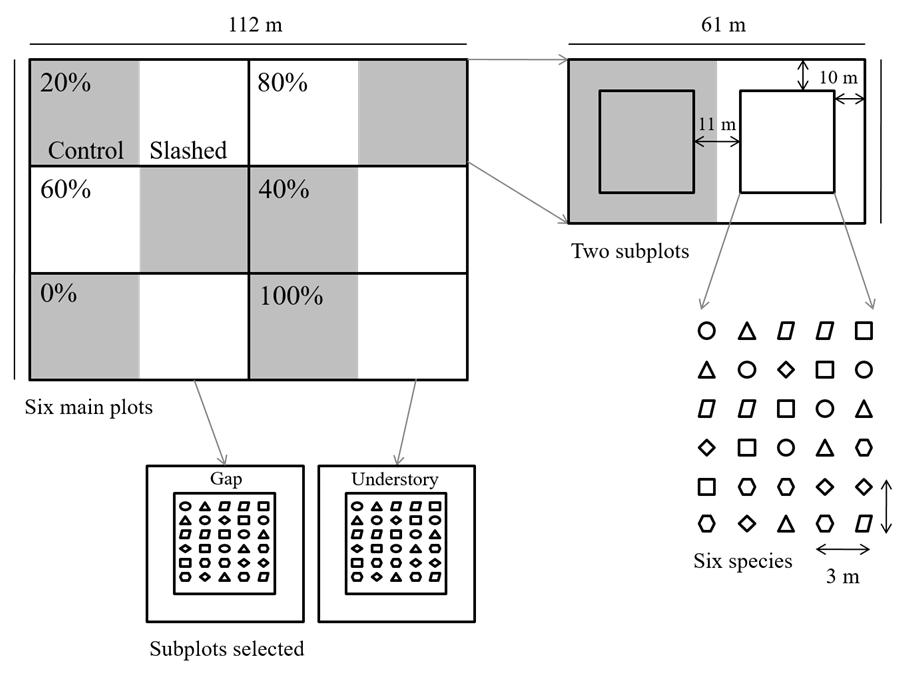


**Figure S1.** Plot and subplot design where silvicultural treatments were applied. The different symbols in the lower right corner are a representation of the six species. White (slashed) subplots represent the experimental units selected for the present study.

**Table S1.** Parameters of fitted regressions to estimate the area of ​​one leaf of each species.

| Species | Range LxW | β0 | β1 | *R^2^* | *P*-value | Std error | DF | F |
| --- | --- | --- | --- | --- | --- | --- | --- | --- |
| *C. fissilis* | 23.1 - 38.0 | -0.01459 | 0.69379 | 0.86 | < 2.2e-16 | 1.095 | 56 | 358.1 |
|  | 38.1 - 56.9 | -0.7383 | 0.71402 | 0.88 | < 2.2e-16 | 1.364 | 77 | 559.8 |
|  | 57.0 - 136.9 | -2.63345 | 0.744736 | 0.97 | < 2.2e-16 | 3.039 | 399 | 1.17E+04 |
|  | 137.0 - 475.3 | 2.766736 | 0.71138 | 0.99 | < 2.2e-16 | 6.533 | 81 | 8358 |
| *T. rosea* | 58.4 - 163.8 | 3.25187 | 0.6376 | 0.94 | < 2.2e-16 | 4.003 | 179 | 2783 |
|  | 163.9 - 361.7 | 5.02413 | 0.64781 | 0.96 | < 2.2e-16 | 7.885 | 161 | 3483 |
| *S. macrophylla* | 16.9 - 112.1 | -2.86202 | 0.72619 | 0.97 | < 2.2e-16 | 2.56 | 291 | 9012 |
|  | 112.2 - 421.9 | 17.05773 | 0.578442 | 0.96 | < 2.2e-16 | 7.706 | 190 | 4.27E+03 |
| *B. excelsa* | 36.5 - 94.1 | 4.12882 | 0.55897 | 0.91 | < 2.2e-16 | 2.465 | 67 | 698.8 |
|  | 94.2 - 178.1 | 4.7726 | 0.5869 | 0.89 | < 2.2e-16 | 4.296 | 158 | 1312 |
|  | 178.2 - 333.8 | -6.2446 | 0.68529 | 0.93 | < 2.2e-16 | 8.654 | 101 | 1289 |
| *C. guianensis* | 29.1 - 91.4 | 0.5227 | 0.66241 | 0.97 | < 2.2e-16 | 1.974 | 88 | 2485 |
|  | 91.5 - 194.8 | -2.9776 | 0.718 | 0.96 | < 2.2e-16 | 4.237 | 177 | 3831 |
|  | 194.9 - 298.2 | 1.1304 | 0.7114 | 0.88 | < 2.2e-16 | 7.588 | 60 | 430.1 |
| *H. courbaril* | 18.6 - 36.5 | 2.46984 | 0.62902 | 0.90 | < 2.2e-16 | 0.8464 | 118 | 1074 |
|  | 36.6 - 99.2 | 0.818776 | 0.703005 | 0.96 | < 2.2e-16 | 1.773 | 673 | 1.83E+04 |
|  | 99.3 - 401.2 | 9.1313 | 0.6032 | 0.97 | < 2.2e-16 | 11.52 | 57 | 1.57E+03 |

**Notes:** Range of length x width measurements used to fit each model, Range LxW; Intercept, β0; Slope, β1; Coefficient of determination, *R^2^*; Significance level, *P*-value; Standard error, Std error; Degrees of freedom, DF; F-value, F. We separated leaves of species into different size classes to adjust regressions. The amount of size classes and the range of size used for each regression for each species are shown in the Range LxW column. We separated leaves into classes of different sizes to ensure statistical presupposes of regressions.

**Table S2.** Pearson correlation matrix of 30 traits of tropical tree saplings growing in Gap.

| Traits | Traits | | | | | | | | | | | | | | | | | | | | | | | | | | | | | |
| --- | --- | --- | --- | --- | --- | --- | --- | --- | --- | --- | --- | --- | --- | --- | --- | --- | --- | --- | --- | --- | --- | --- | --- | --- | --- | --- | --- | --- | --- | --- |
|  | *MCD* | *CPA* | *CL* | *CR* | *CLR* | *RCL* | *TLA* | *LAI* | *LA* | *SLA* | *LDMC* | *N*_mass_ | *P*_mass_ | *K*_mass_ | *Chl a* | *Chl b* | *Car*_c+x_ | *Chl a*+*b* | *Chl a*/*b* | *F*_V_/*F*_M_ | *PI*_ABS_ | *PI*_total_ | *A*_max_ | *R*_d_ | *g*_s_ | *E* | *CUE* | *PNUE* | *PPUE* | *PKUE* |
| *MCD* |  |  |  |  |  |  |  |  |  |  |  |  |  |  |  |  |  |  |  |  |  |  |  |  |  |  |  |  |  |  |
| *CPA* | **0.98** |  |  |  |  |  |  |  |  |  |  |  |  |  |  |  |  |  |  |  |  |  |  |  |  |  |  |  |  |  |
| *CL* | **0.44** | **0.44** |  |  |  |  |  |  |  |  |  |  |  |  |  |  |  |  |  |  |  |  |  |  |  |  |  |  |  |  |
| *CR* | **0.65** | **0.63** | 0.16 |  |  |  |  |  |  |  |  |  |  |  |  |  |  |  |  |  |  |  |  |  |  |  |  |  |  |  |
| *CLR* | **0.35** | **0.37** | **0.80** | **0.41** |  |  |  |  |  |  |  |  |  |  |  |  |  |  |  |  |  |  |  |  |  |  |  |  |  |  |
| *RCL* | **-0.23** | **-0.20** | **0.72** | ***-0.29*** | **0.62** |  |  |  |  |  |  |  |  |  |  |  |  |  |  |  |  |  |  |  |  |  |  |  |  |  |
| *TLA* | **0.53** | **0.53** | **0.79** | 0.01 | **0.44** | **0.43** |  |  |  |  |  |  |  |  |  |  |  |  |  |  |  |  |  |  |  |  |  |  |  |  |
| *LAI* | **-0.45** | **-0.40** | **0.35** | **-0.60** | 0.15 | **0.74** | **0.40** |  |  |  |  |  |  |  |  |  |  |  |  |  |  |  |  |  |  |  |  |  |  |  |
| *LA* | -0.08 | -0.06 | 0.06 | **-0.21** | -0.18 | **0.22** | ***0.27*** | **0.41** |  |  |  |  |  |  |  |  |  |  |  |  |  |  |  |  |  |  |  |  |  |  |
| *SLA* | ***0.32*** | ***0.30*** | **-0.21** | **0.37** | -0.04 | **-0.45** | -0.14 | **-0.48** | **-0.41** |  |  |  |  |  |  |  |  |  |  |  |  |  |  |  |  |  |  |  |  |  |
| *LDMC* | -0.13 | -0.13 | 0.08 | 0.10 | 0.15 | 0.18 | -0.04 | 0.08 | **0.19** | **-0.35** |  |  |  |  |  |  |  |  |  |  |  |  |  |  |  |  |  |  |  |  |
| *N*_mass_ | **0.60** | **0.54** | 0.05 | **0.52** | 0.10 | **-0.37** | 0.13 | **-0.49** | **-0.19** | **0.43** | -0.14 |  |  |  |  |  |  |  |  |  |  |  |  |  |  |  |  |  |  |  |
| *P*_mass_ | **0.40** | **0.36** | -0.03 | ***0.32*** | 0.01 | ***-0.32*** | 0.01 | **-0.43** | ***-0.32*** | **0.42** | -0.17 | **0.68** |  |  |  |  |  |  |  |  |  |  |  |  |  |  |  |  |  |  |
| *K*_mass_ | 0.00 | -0.04 | **-0.49** | -0.05 | **-0.54** | **-0.53** | ***-0.27*** | ***-0.32*** | -0.02 | 0.14 | -0.11 | ***0.32*** | **0.39** |  |  |  |  |  |  |  |  |  |  |  |  |  |  |  |  |  |
| *Chl a* | **0.36** | **0.36** | **0.41** | **0.43** | **0.45** | **0.19** | **0.24** | -0.08 | 0.01 | 0.05 | 0.14 | **0.33** | -0.01 | ***-0.29*** |  |  |  |  |  |  |  |  |  |  |  |  |  |  |  |  |
| *Chl b* | ***0.29*** | ***0.28*** | **0.36** | ***0.27*** | **0.37** | **0.21** | ***0.25*** | 0.03 | 0.03 | 0.01 | 0.07 | ***0.27*** | -0.10 | ***-0.27*** | **0.93** |  |  |  |  |  |  |  |  |  |  |  |  |  |  |  |
| *Car*_c+x_ | ***0.25*** | ***0.24*** | ***0.28*** | ***0.32*** | ***0.30*** | 0.15 | 0.17 | -0.02 | 0.06 | 0.01 | 0.12 | ***0.26*** | -0.08 | **-0.22** | **0.82** | **0.88** |  |  |  |  |  |  |  |  |  |  |  |  |  |  |
| *Chl a*+*b* | **0.35** | **0.34** | **0.40** | **0.40** | **0.43** | **0.19** | ***0.24*** | -0.06 | 0.02 | 0.04 | 0.12 | ***0.32*** | -0.03 | ***-0.29*** | **1.00** | **0.96** | **0.85** |  |  |  |  |  |  |  |  |  |  |  |  |  |
| *Chl a*/*b* | 0.14 | 0.15 | 0.11 | **0.39** | **0.20** | -0.03 | -0.08 | ***-0.30*** | -0.06 | 0.09 | 0.18 | 0.12 | ***0.25*** | -0.04 | **0.20** | -0.16 | -0.12 | 0.12 |  |  |  |  |  |  |  |  |  |  |  |  |
| *F*_V_/*F*_M_ | -0.04 | -0.04 | **0.21** | -0.12 | 0.08 | ***0.27*** | 0.17 | 0.15 | 0.13 | **-0.21** | 0.17 | -0.12 | -0.09 | **-0.22** | **0.22** | **0.22** | 0.16 | **0.22** | 0.06 |  |  |  |  |  |  |  |  |  |  |  |
| *PI*_ABS_ | -0.07 | -0.08 | **0.36** | **-0.41** | 0.10 | **0.45** | **0.37** | **0.44** | 0.17 | **-0.43** | 0.01 | -0.16 | -0.18 | **-0.19** | 0.06 | **0.19** | 0.08 | 0.09 | ***-0.26*** | **0.67** |  |  |  |  |  |  |  |  |  |  |
| *PI*_total_ | **0.21** | 0.16 | **0.46** | -0.08 | ***0.26*** | ***0.30*** | **0.39** | 0.13 | -0.12 | ***-0.31*** | -0.05 | **0.20** | 0.09 | -0.12 | ***0.25*** | ***0.27*** | 0.17 | ***0.25*** | -0.01 | **0.39** | **0.65** |  |  |  |  |  |  |  |  |  |
| *A*_max_ | ***0.31*** | ***0.29*** | ***0.25*** | ***0.29*** | ***0.25*** | -0.02 | 0.16 | **-0.21** | -0.13 | 0.06 | -0.04 | **0.41** | **0.32** | 0.03 | **0.21** | 0.11 | 0.00 | **0.19** | **0.24** | 0.14 | 0.07 | **0.37** |  |  |  |  |  |  |  |  |
| *R*_d_ | -0.06 | -0.07 | 0.00 | -0.02 | 0.08 | -0.01 | -0.08 | -0.06 | -0.15 | -0.08 | 0.11 | 0.02 | 0.03 | 0.09 | -0.04 | -0.09 | **-0.19** | -0.06 | 0.18 | -0.17 | 0.02 | 0.07 | **0.22** |  |  |  |  |  |  |  |
| *g*_s_ | 0.13 | 0.14 | 0.10 | 0.04 | 0.03 | 0.00 | 0.18 | 0.01 | 0.06 | 0.01 | -0.04 | **0.19** | 0.09 | -0.05 | -0.04 | -0.01 | -0.10 | -0.04 | -0.15 | **0.24** | ***0.26*** | **0.23** | **0.66** | 0.03 |  |  |  |  |  |  |
| *E* | 0.13 | 0.13 | 0.10 | 0.09 | 0.09 | -0.03 | 0.12 | -0.07 | 0.02 | 0.03 | -0.08 | 0.17 | 0.08 | -0.06 | 0.02 | 0.03 | -0.03 | 0.02 | -0.04 | **0.19** | **0.24** | **0.24** | **0.58** | 0.09 | **0.75** |  |  |  |  |  |
| *CUE* | ***0.25*** | **0.23** | 0.13 | **0.24** | 0.07 | -0.06 | 0.12 | -0.15 | 0.04 | 0.10 | -0.04 | ***0.27*** | 0.18 | -0.07 | 0.18 | 0.14 | 0.18 | 0.18 | 0.03 | **0.21** | -0.02 | 0.14 | **0.47** | **-0.65** | **0.38** | ***0.30*** |  |  |  |  |
| *PNUE* | 0.01 | 0.03 | 0.05 | 0.09 | 0.12 | -0.02 | -0.04 | -0.11 | **-0.23** | ***0.31*** | -0.14 | -0.12 | 0.04 | -0.13 | -0.02 | -0.10 | **-0.19** | -0.04 | **0.22** | 0.10 | -0.07 | 0.03 | **0.70** | **0.21** | **0.50** | **0.47** | ***0.27*** |  |  |  |
| *PPUE* | 0.12 | 0.12 | 0.12 | **0.23** | **0.20** | -0.02 | 0.02 | -0.11 | -0.12 | **0.21** | -0.04 | 0.04 | ***-0.27*** | -0.18 | **0.21** | 0.17 | 0.06 | **0.21** | 0.08 | 0.06 | -0.02 | 0.09 | **0.69** | 0.17 | **0.54** | **0.47** | ***0.31*** | **0.79** |  |  |
| *PKUE* | **0.23** | ***0.25*** | **0.41** | **0.26** | **0.48** | **0.23** | **0.22** | -0.04 | **-0.22** | ***0.27*** | -0.07 | 0.07 | 0.03 | **-0.68** | **0.33** | **0.24** | 0.13 | ***0.31*** | **0.24** | **0.21** | 0.02 | 0.15 | **0.52** | 0.03 | **0.35** | ***0.32*** | **0.34** | **0.62** | **0.56** |  |

**Notes:** Correlations with P value < 0.001 are shown in bold, P < 0.01 in bold and italic, P < 0.05 in bold and underlined. Correlations that are not in bold are not significant. For abbreviations, see Table 1 in the section Results.

**Table S3.** Pearson correlation matrix of 30 traits of tropical tree saplings growing in Understory

| Traits | Traits | | | | | | | | | | | | | | | | | | | | | | | | | | | | | |
| --- | --- | --- | --- | --- | --- | --- | --- | --- | --- | --- | --- | --- | --- | --- | --- | --- | --- | --- | --- | --- | --- | --- | --- | --- | --- | --- | --- | --- | --- | --- |
|  | *MCD* | *CPA* | *CL* | *CR* | *CLR* | *RCL* | *TLA* | *LAI* | *LA* | *SLA* | *LDMC* | *N*_mass_ | *P*_mass_ | *K*_mass_ | *Chl a* | *Chl b* | *Car*_c+x_ | *Chl a*+*b* | *Chl a*/*b* | *F*_V_/*F*_M_ | *PI*_ABS_ | *PI*_total_ | *A*_max_ | *R*_d_ | *g*_s_ | *E* | *CUE* | *PNUE* | *PPUE* | *PKUE* |
| *MCD* |  |  |  |  |  |  |  |  |  |  |  |  |  |  |  |  |  |  |  |  |  |  |  |  |  |  |  |  |  |  |
| *CPA* | **0.94** |  |  |  |  |  |  |  |  |  |  |  |  |  |  |  |  |  |  |  |  |  |  |  |  |  |  |  |  |  |
| *CL* | 0.06 | 0.04 |  |  |  |  |  |  |  |  |  |  |  |  |  |  |  |  |  |  |  |  |  |  |  |  |  |  |  |  |
| *CR* | **0.38** | ***0.30*** | 0.19 |  |  |  |  |  |  |  |  |  |  |  |  |  |  |  |  |  |  |  |  |  |  |  |  |  |  |  |
| *CLR* | **-0.25** | -0.20 | **0.83** | 0.14 |  |  |  |  |  |  |  |  |  |  |  |  |  |  |  |  |  |  |  |  |  |  |  |  |  |  |
| *RCL* | **-0.41** | ***-0.33*** | **0.80** | -0.01 | **0.91** |  |  |  |  |  |  |  |  |  |  |  |  |  |  |  |  |  |  |  |  |  |  |  |  |  |
| *TLA* | **0.82** | **0.83** | **0.23** | 0.09 | -0.07 | -0.16 |  |  |  |  |  |  |  |  |  |  |  |  |  |  |  |  |  |  |  |  |  |  |  |  |
| *LAI* | -0.20 | -0.15 | ***0.32*** | **-0.43** | ***0.33*** | **0.44** | ***0.30*** |  |  |  |  |  |  |  |  |  |  |  |  |  |  |  |  |  |  |  |  |  |  |  |
| *LA* | 0.12 | 0.08 | 0.12 | ***-0.28*** | -0.01 | 0.04 | ***0.33*** | **0.42** |  |  |  |  |  |  |  |  |  |  |  |  |  |  |  |  |  |  |  |  |  |  |
| *SLA* | **0.42** | **0.38** | **-0.47** | **0.22** | **-0.55** | **-0.57** | **0.21** | ***-0.28*** | **-0.23** |  |  |  |  |  |  |  |  |  |  |  |  |  |  |  |  |  |  |  |  |  |
| *LDMC* | **-0.46** | **-0.37** | **0.23** | -0.15 | **0.41** | **0.45** | ***-0.32*** | 0.16 | **0.27** | **-0.73** |  |  |  |  |  |  |  |  |  |  |  |  |  |  |  |  |  |  |  |  |
| *N*_mass_ | ***0.34*** | ***0.31*** | -0.05 | **0.42** | -0.13 | -0.16 | **0.22** | -0.17 | -0.16 | **0.54** | **-0.39** |  |  |  |  |  |  |  |  |  |  |  |  |  |  |  |  |  |  |  |
| *P*_mass_ | 0.13 | 0.13 | -0.12 | 0.10 | **-0.21** | -0.15 | 0.08 | -0.10 | 0.04 | **0.37** | **-0.36** | **0.36** |  |  |  |  |  |  |  |  |  |  |  |  |  |  |  |  |  |  |
| *K*_mass_ | 0.17 | 0.11 | -0.16 | 0.16 | -0.20 | -0.16 | 0.09 | -0.04 | -0.07 | ***0.35*** | **-0.39** | **0.51** | **0.55** |  |  |  |  |  |  |  |  |  |  |  |  |  |  |  |  |  |
| *Chl a* | **0.26** | **0.25** | 0.10 | **0.40** | 0.05 | -0.01 | **0.21** | -0.02 | 0.00 | 0.11 | -0.06 | **0.51** | -0.04 | 0.20 |  |  |  |  |  |  |  |  |  |  |  |  |  |  |  |  |
| *Chl b* | ***0.31*** | ***0.28*** | 0.06 | **0.38** | 0.00 | -0.08 | **0.24** | -0.04 | -0.03 | 0.17 | -0.14 | **0.49** | -0.09 | 0.19 | **0.97** |  |  |  |  |  |  |  |  |  |  |  |  |  |  |  |
| *Car*_c+x_ | ***0.34*** | ***0.33*** | 0.10 | ***0.35*** | 0.02 | -0.05 | ***0.28*** | -0.05 | -0.01 | 0.11 | -0.10 | **0.39** | -0.12 | 0.12 | **0.91** | **0.93** |  |  |  |  |  |  |  |  |  |  |  |  |  |  |
| *Chl a*+*b* | ***0.29*** | ***0.27*** | 0.09 | **0.39** | 0.03 | -0.04 | **0.23** | -0.04 | -0.01 | 0.14 | -0.10 | **0.51** | -0.04 | 0.19 | **0.98** | **0.98** | **0.89** |  |  |  |  |  |  |  |  |  |  |  |  |  |
| *Chl a*/*b* | **-0.22** | **-0.14** | 0.06 | 0.03 | 0.12 | 0.18 | -0.15 | 0.07 | 0.16 | **-0.24** | ***0.35*** | -0.03 | 0.18 | 0.01 | 0.10 | -0.14 | -0.13 | -0.01 |  |  |  |  |  |  |  |  |  |  |  |  |
| *F*_V_/*F*_M_ | ***-0.36*** | ***-0.35*** | **0.26** | -0.07 | ***0.35*** | **0.43** | **-0.25** | 0.18 | **0.26** | **-0.59** | **0.48** | -0.07 | 0.00 | 0.07 | 0.10 | 0.01 | 0.00 | 0.06 | **0.40** |  |  |  |  |  |  |  |  |  |  |  |
| *PI*_ABS_ | -0.13 | -0.18 | ***0.31*** | -0.09 | **0.26** | ***0.29*** | -0.05 | 0.17 | ***0.31*** | **-0.54** | ***0.35*** | 0.05 | ***-0.30*** | -0.02 | ***0.35*** | ***0.29*** | ***0.32*** | ***0.31*** | 0.20 | **0.60** |  |  |  |  |  |  |  |  |  |  |
| *PI*_total_ | -0.05 | -0.12 | ***0.33*** | -0.07 | 0.20 | ***0.28*** | 0.01 | 0.11 | ***0.28*** | **-0.46** | ***0.33*** | 0.11 | -0.19 | -0.04 | **0.27** | **0.20** | **0.23** | **0.24** | **0.22** | **0.48** | **0.81** |  |  |  |  |  |  |  |  |  |
| *A*_max_ | -0.01 | -0.08 | ***0.29*** | 0.19 | **0.21** | **0.24** | -0.01 | 0.02 | **0.23** | **-0.21** | ***0.28*** | **0.38** | 0.08 | 0.20 | ***0.35*** | 0.24 | **0.23** | ***0.30*** | ***0.33*** | **0.38** | **0.48** | **0.68** |  |  |  |  |  |  |  |  |
| *R*_d_ | -0.01 | 0.05 | 0.12 | 0.02 | 0.16 | 0.16 | 0.05 | 0.14 | -0.06 | -0.13 | 0.10 | 0.02 | **-0.21** | 0.03 | 0.17 | 0.20 | **0.26** | 0.14 | -0.11 | -0.05 | 0.06 | -0.03 | 0.02 |  |  |  |  |  |  |  |
| *g*_s_ | -0.02 | -0.01 | 0.00 | -0.10 | -0.04 | 0.07 | 0.01 | 0.01 | ***0.29*** | -0.03 | **0.23** | 0.14 | 0.12 | 0.09 | 0.10 | 0.01 | 0.04 | 0.05 | ***0.27*** | 0.12 | 0.14 | ***0.36*** | **0.56** | -0.07 |  |  |  |  |  |  |
| *E* | 0.02 | 0.02 | -0.06 | -0.04 | -0.11 | -0.02 | 0.02 | -0.03 | ***0.32*** | 0.03 | 0.19 | 0.13 | 0.24 | 0.17 | 0.02 | -0.08 | -0.06 | -0.03 | ***0.33*** | 0.09 | 0.09 | ***0.32*** | **0.53** | -0.20 | **0.89** |  |  |  |  |  |
| *CUE* | 0.02 | -0.03 | 0.15 | 0.04 | 0.04 | 0.10 | 0.02 | -0.03 | ***0.29*** | -0.07 | 0.15 | 0.16 | 0.15 | -0.06 | 0.07 | 0.00 | -0.03 | 0.07 | **0.22** | 0.18 | **0.22** | **0.49** | **0.57** | **-0.64** | **0.43** | **0.45** |  |  |  |  |
| *PNUE* | **0.25** | **0.13** | **-0.22** | 0.13 | ***-0.34*** | ***-0.29*** | 0.11 | -0.15 | 0.13 | **0.47** | ***-0.34*** | **0.37** | **0.37** | **0.39** | 0.17 | 0.13 | 0.09 | 0.15 | 0.11 | -0.08 | -0.04 | 0.20 | **0.57** | -0.10 | **0.41** | **0.47** | **0.39** |  |  |  |
| *PPUE* | **0.23** | **0.12** | -0.09 | 0.13 | -0.19 | -0.20 | 0.13 | -0.09 | 0.02 | ***0.31*** | -0.12 | **0.40** | ***-0.31*** | 0.07 | ***0.32*** | ***0.32*** | ***0.28*** | ***0.31*** | -0.04 | -0.15 | 0.17 | **0.33** | **0.53** | 0.15 | ***0.32*** | **0.27** | ***0.22*** | **0.63** |  |  |
| *PKUE* | 0.10 | 0.06 | -0.05 | 0.10 | -0.12 | -0.11 | 0.02 | -0.12 | 0.07 | **0.26** | 0.01 | 0.11 | **-0.21** | **-0.54** | 0.02 | -0.01 | 0.00 | 0.02 | 0.04 | **-0.25** | -0.06 | 0.16 | **0.26** | -0.13 | **0.21** | **0.21** | **0.39** | **0.39** | **0.54** |  |

**Notes:** Correlations with P value < 0.001 are shown in bold, P < 0.01 in bold and italic, P < 0.05 in bold and underlined. Correlations that are not in bold are not significant. For abbreviations, see Table 1 in the section Results.

**Table S2**. Results of the mixed effect models examining the effects of light environment on the relations between growth and traits.

| Trait | Factors effect | | | Slope ± error | | *R^2^*_m_ | *R^2^*_c_ | Random effect |
| --- | --- | --- | --- | --- | --- | --- | --- | --- |
|  | Environment | Trait | Interaction | *Gap* | *Understory* |  |  |  |
| ln *MCD* | *** | *** | n.s. | 0.437 ± 0.064 | 0.560 ± 0.263 | 0.77 | 0.81 | Species |
| ln *CPA* | *** | *** | n.s. | 0.218 ± 0.032 | 0.273 ± 0.130 | 0.77 | 0.81 | Species |
| *CL* | *** | *** | ** | 0.454 ± 0.065 | 0.690 ± 0.237 | 0.82 | 0.94 | Species + Plots |
| *CR* | *** | n.s. | n.s. | -0.001 ± 0.097 | -0.002 ± 0.203 | 0.78 | 0.84 | Species + Plots |
| *CLR* | *** | *** | ** | 1.207 ± 0.482 | 0.543 ± 0.919 | 0.84 | 0.92 | Species |
| *RCL* | *** | *** | * | 0.352 ± 0.193 | 0.106 ± 0.415 | 0.78 | 0.86 | Species + Plots |
| ln *TLA* | *** | *** | * | 0.169 ± 0.025 | 0.279 ± 0.119 | 0.76 | 0.77 | Species |
| *LAI* | *** | n.s. | n.s. | 0.032 ± 0.050 | 0.084 ± 0.368 | 0.78 | 0.84 | Species + Plots |
| *LA* | *** | ** | * | 0.003 ± 0.002 | 0.006 ± 0.005 | 0.85 | 0.93 | Species |
| *SLA* | *** | ** | * | -0.008 ± 0.006 | -0.001 ± 0.011 | 0.76 | 0.86 | Species + Plots |
| *LDMC* | *** | n.s. | n.s. | -0.001 ± 0.001 | 8.9*10^-5^ ± 0.003 | 0.87 | 0.91 | Species |
| *N*_mass_ | ** | *** | *** | 0.054 ± 0.028 | -0.007 ± 0.063 | 0.87 | 0.91 | Species |
| *P*_mass_ | *** | n.s. | n.s. | 0.077 ± 0.522 | -0.083 ± 1.091 | 0.78 | 0.84 | Species + Plots |
| *K*_mass_ | *** | n.s. | n.s. | -0.028 ± 0.029 | -0.009 ± 0.063 | 0.87 | 0.91 | Species |
| *Chl a* | *** | *** | ** | 0.343 ± 0.185 | 0.003 ± 0.389 | 0.87 | 0.91 | Species |
| *Chl b* | *** | *** | ** | 1.134 ± 0.566 | 0.070 ± 1.185 | 0.80 | 0.86 | Species + Plots |
| *Car* _c+x_ | *** | ** | * | 0.740 ± 0.509 | 0.101 ± 1.083 | 0.79 | 0.85 | Species + Plots |
| *Chl* *a*+*b* | *** | *** | ** | 0.272 ± 0.142 | 0.008 ± 0.298 | 0.87 | 0.91 | Species |
| *Chl* *a*/*b* | *** | * | n.s. | -0.225 ± 0.206 | -0.152 ± 0.529 | 0.79 | 0.84 | Species + Plots |
| *F*_V_/*F*_M_ | n.s. | n.s. | n.s. | 1.768 ± 2.747 | -0.416 ± 10.186 | 0.78 | 0.84 | Species + Plots |
| *PI*_ABS_ | *** | *** | * | 0.192 ± 0.066 | 0.083 ± 0.181 | 0.81 | 0.88 | Species + Plots |
| *PI*_total_ | *** | *** | n.s. | 0.380 ± 0.105 | 0.338 ± 0.365 | 0.82 | 0.90 | Species + Plots |
| *A*_max_ | *** | ** | n.s. | 0.035 ± 0.025 | 0.032 ± 0.059 | 0.86 | 0.92 | Species |
| *R*_d_ | *** | n.s. | n.s. | -0.020 ± 0.169 | -0.005 ± 0.439 | 0.78 | 0.84 | Species + Plots |
| *g*_s_ | *** | * | n.s. | 0.801 ± 0.698 | 0.436 ± 1.833 | 0.78 | 0.84 | Species + Plots |
| *E* | *** | n.s. | n.s. | 0.042 ± 0.060 | 0.025 ± 0.150 | 0.78 | 0.84 | Species + Plots |
| *CUE* | *** | n.s. | n.s. | 0.020 ± 0.023 | 0.005 ± 0.045 | 0.77 | 0.84 | Species + Plots |
| *PNUE* | *** | n.s. | n.s. | -0.001 ± 0.002 | 0.001 ± 0.005 | 0.78 | 0.84 | Species + Plots |
| *PPUE* | *** | n.s. | n.s. | 1.9*10^-5^ ± 4.5*10^-5^ | 2.3*10^-5^ ± 9.7*10^-5^ | 0.78 | 0.84 | Species + Plots |
| *PKUE* | * | *** | * | 2.8*10^-4^ ± 2.5*10^-4^ | -2.3*10^-5^ ± 0.00l1 | 0.87 | 0.81 | Species |

**Notes:** Random effects of the best models are controlled for intercepts of species and plots. Coefficients of determination marginal (*R^2^*_m_) and conditional (*R^2^*_c_). P values of model factor effects are * *P* < 0.05, ** *P* < 0.01, *** *P* < 0.001, and not significant (n.s.) *P* ≥ 0.05. For abbreviations, see Table 1 in the section Results.


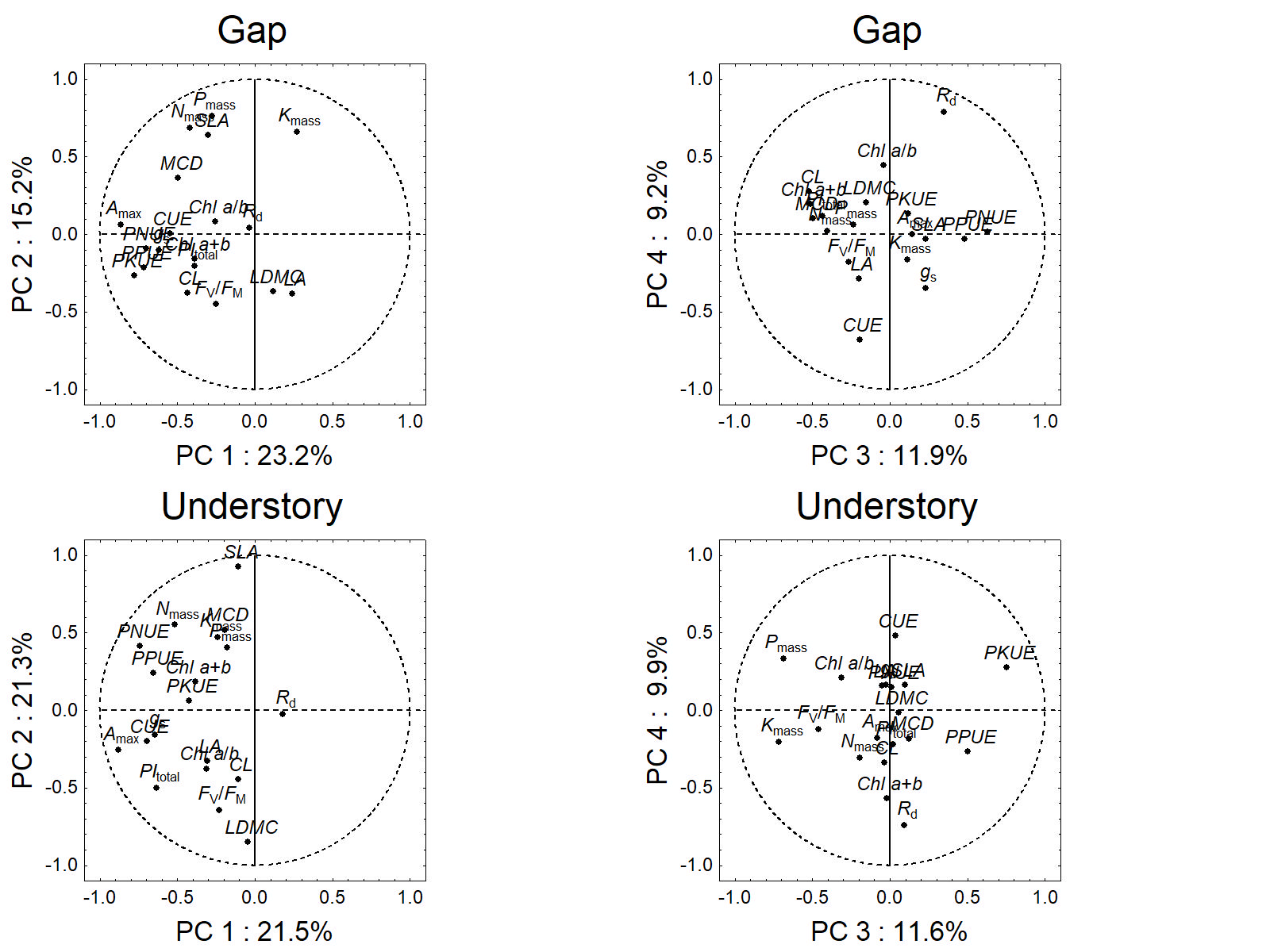


**Figure S2.** First four axes of principal component analysis of 19 crown and leaf traits (general PCA) of tropical tree saplings growing in two contrasting light environments (Gap and Understory). **Set of traits used:** *MCD, CL, SLA, LA, LDMC, N*_mass_, *P*_mass_, *K*_mass_, *Chl* *a*+*b*, *Chl* *a*/*b*, *F*_V_/*F*_M_, *PI*_total_, *A*_max_, *R*_d_, *g*_s_, *CUE*, *PNUE*, *PPUE*, *PKUE*. **We removed:** *CPA* and *CR*, which derive from *MCD* and represent the horizontal area of light interception, as *MCD*; *CLR, RCL,* and *LAI*, which represent crown depth, as *CL*; *TLA*, which is strongly related to *CL* (in Gap) and *MCD* (in Understory); *Chl a, Chl b*, and *Car* _c+x_, which are strongly related to *Chl* *a*+*b*; and *PI*_ABS_, which is strongly related to *PI*_total_ in Understory. For abbreviations, see Table 1 in the section Results.


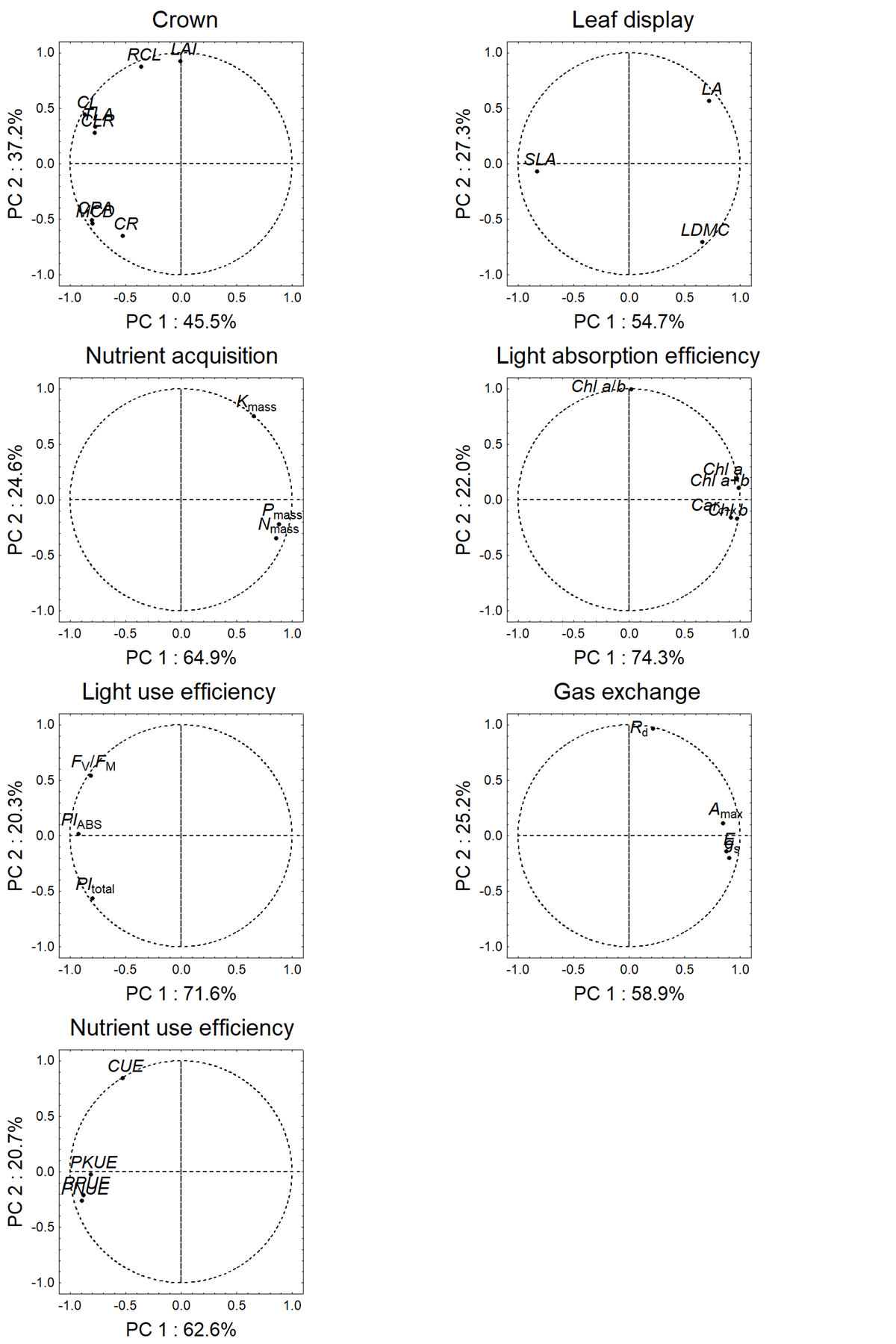


**Figure S3.** First two axes of seven principal component analysis performed for different trait groups (categorized PCAs) of tropical tree saplings growing in Gap. For abbreviations, see Table 1 in the section Results.


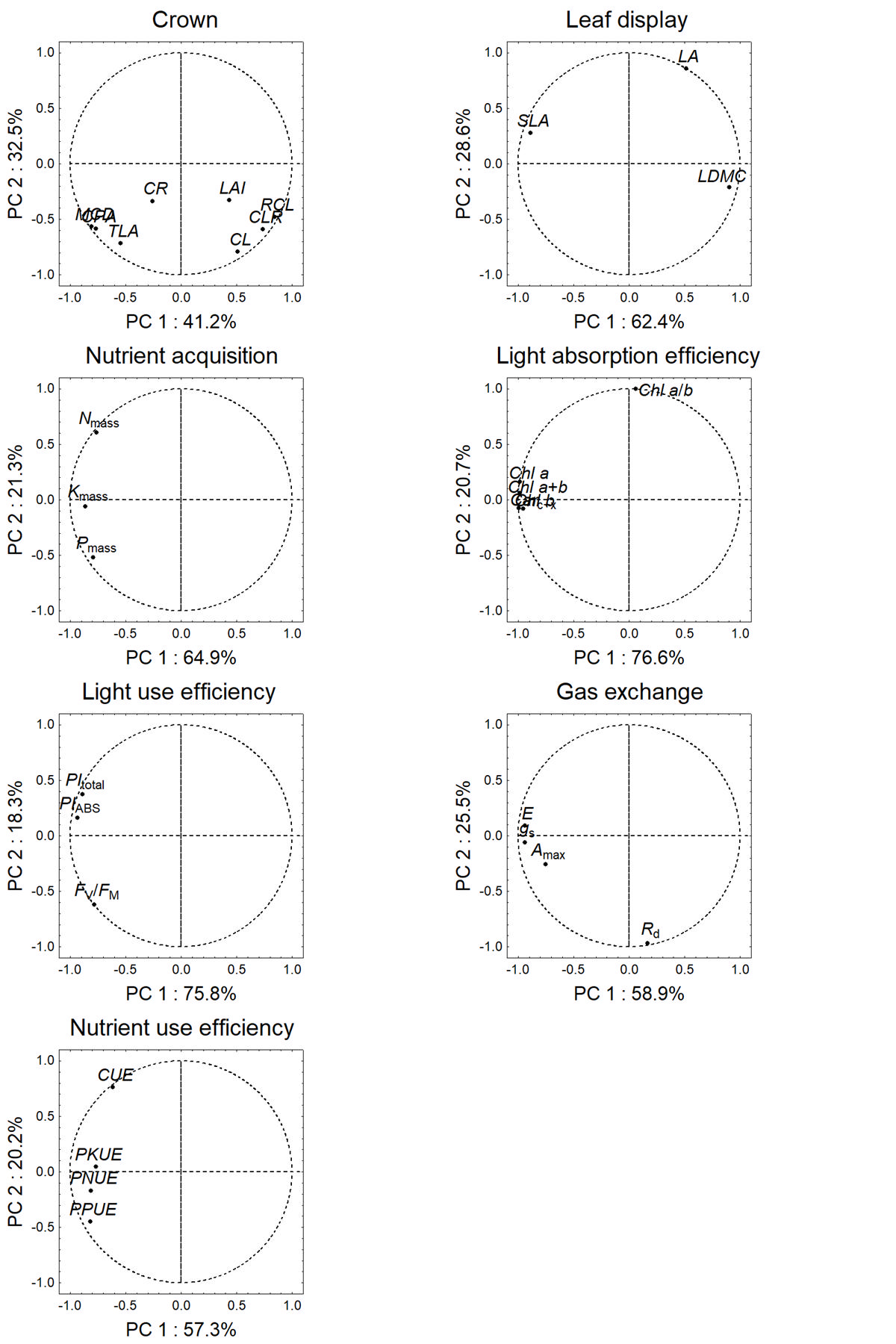


**Figure S4.** First two axes of seven principal component analysis performed for different traits groups (categorized PCAs) of tropical tree saplings growing in Understory. For abbreviations, see Table 1 in the section Results.

**Table S3.** Eigenvalues and main loads of the four first axis of the general PCA in two light environments.

| Axis | Eigenvalue | Traits with the highest loadings | | | |
| --- | --- | --- | --- | --- | --- |
| **Gap** | | | | | |
| PC1 | 23.2% | *A*_max_ = -0.87 | *PKUE* = -0.78 | *PPUE* = -0.72 | *PNUE* = -0.70 |
| PC2 | 15.2% | *P*_mass_ = -0.77 | *N*_mass_ = -0.69 | *K*_mass_ = -0.66 | *SLA* = -0.64 |
| PC3 | 11.9% | *PNUE* = 0.63 |  |  |  |
| PC4 | 9.2% | *R*_d_ = -0.79 | *CUE* = 0.68 |  |  |
| **Understory** | | | | | |
| PC1 | 21.5% | *A*_max_ = -0.88 | *PNUE* = -0.74 | *PPUE* = -0.65 | *PI*_total_ = -0.64 |
| PC2 | 21.3% | *SLA* = 0.83 | *LDMC* = -0.85 |  |  |
| PC3 | 11.6% | *PKUE* = -0.75 | *K*_mass_ = 0.71 | *P*_mass_ = 0.69 |  |
| PC4 | 9.9% | *R*_d_ = 0.74 |  |  |  |

**Notes:** For abbreviations, see Table 1 in the section Results.

**Table S4.** Eigenvalues and highest loadings of the two first axes of the categorized PCAs in two light environments.

|  | Crown | | Leaf display | | Nutrient acquisition | | Light absorption | | Light use | | Gas exchange | | Nutrient use | |
| --- | --- | --- | --- | --- | --- | --- | --- | --- | --- | --- | --- | --- | --- | --- |
|  | PC1 | PC2 | PC1 | PC2 | PC1 | PC2 | PC1 | PC2 | PC1 | PC2 | PC1 | PC2 | PC1 | PC2 |
|  | Gap | | | | | | | | | | | | | |
| Eigenvalues (%) | 45.5 | 37.2 | 54.7 | 27.3 | 64.9 | 24.6 | 74.3 | 22.0 | 71.6 | 20.3 | 58.9 | 25.2 | 62.6 | 20.7 |
| Traits | *CL* | *LAI* | *SLA* | *LDMC* | *P*_mass_ | *K*_mass_ | *Chl a*+*b* | *Chl a*/*b* | *PI*_ABS_ | *PI*_total_ | *g*_s_ | *R*_d_ | *PNUE* | *CUE* |
| Loadings | -0.87 | -0.92 | 0.83 | -0.70 | 0.89 | 0.75 | -0.99 | -1.00 | -0.92 | 0.56 | -0.90 | -0.97 | -0.89 | 0.84 |
|  | Understory | | | | | | | | | | | | | |
| Eigenvalues (%) | 41.2 | 32.5 | 62.4 | 28.6 | 64.9 | 21.3 | 76.6 | 20.7 | 75.8 | 18.3 | 59.9 | 25.5 | 57.3 | 20.2 |
| Traits | *RCL* | *CL* | *LDMC* | *LA* | *K*_mass_ | *N*_mass_ | *Chl b* | *Chl* *a*/*b* | *PI*_ABS_ | *F*_V_/*F*_M_ | *g*_s_ | *R*_d_ | *PPUE* | *CUE* |
| Loadings | 0.84 | 0.78 | -0.90 | -0.86 | 0.86 | 0.60 | 0.99 | 1.00 | 0.93 | 0.62 | -0.94 | -0.97 | -0.82 | 0.76 |

**Notes:** For abbreviations, see Table 1 in the section Results.


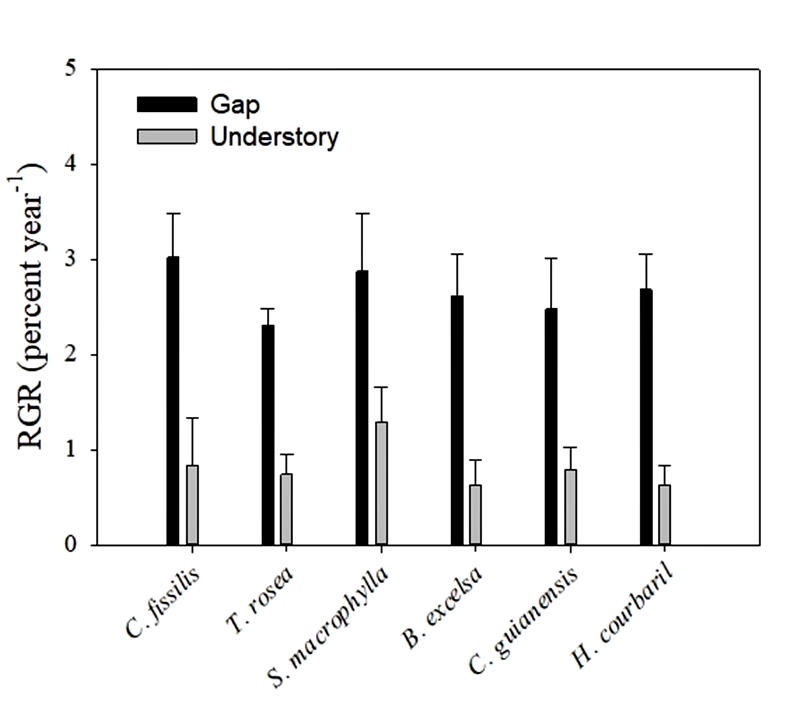


**Figure S5.** Relative growth rate of saplings of the six tropical tree species, two years after planting in two light environments. Mean values ± standard deviation.
